# Involving members of vulnerable populations in the development of patient decision aids: a mixed methods sequential explanatory study

**DOI:** 10.1186/s12911-016-0399-8

**Published:** 2017-01-19

**Authors:** Michèle Dugas, Marie-Ève Trottier, Selma Chipenda Dansokho, Gratianne Vaisson, Thierry Provencher, Heather Colquhoun, Maman Joyce Dogba, Sophie Dupéré, Angela Fagerlin, Anik M. C. Giguere, Lynne Haslett, Aubri S. Hoffman, Noah M. Ivers, France Légaré, Jean Légaré, Carrie A. Levin, Matthew Menear, Jean-Sébastien Renaud, Dawn Stacey, Robert J. Volk, Holly O. Witteman

**Affiliations:** 1 Office of Education and Professional Development, Faculty of Medicine, Laval University, 1050 avenue de la Médecine, Quebec City, QC G1V 0A6 Canada; 2 Research Centre of the CHU de Québec, 1050 avenue de la Médecine, Quebec City, QC G1V 0A6 Canada; 3 Department of Occupational Science and Occupational Therapy, Faculty of Medicine, University of Toronto, 160-500 University Ave, Toronto, ON M5G 1V7 Canada; 4 Department of Family and Emergency Medicine, Faculty of Medicine, Laval University, 1050 avenue de la Médecine, Quebec City, QC G1V 0A6 Canada; 5 Faculty of Nursing, Laval University, 1050 avenue de la Médecine, Quebec City, QC G1V 0A6 Canada; 6 Department of Population Health Sciences, University of Utah, 295 Chipeta Way, Williams Building, Room 1C448, Salt Lake City, UT 84132 USA; 7 Quebec Centre for Excellence on Aging, Research Centre of the CHU de Quebec, St-Sacrement Hospital, 1050, chemin Ste-Foy, Quebec City, QC G1S 4L8 Canada; 8 East End Community Health Centre, 1619 Queen Street East, Toronto, ON M4L 1G4 Canada; 9 Department of Health Services Research, The MD Anderson Cancer Center, FCT9.5028, 1400 Pressler Street, Houston, TX 77030 USA; 10 Family Practice Health Centre, Institute for Health Systems Solutions and Virtual Care and Women’s College Research Institute, Women’s College Hospital, 76 Grenville St, Toronto, ON M5S1B2 Canada; 11 Department of Family and Community Medicine, Institute of Health Policy, Management and Evaluation, University of Toronto, 500 University Ave, Toronto, ON M5G1V7 Canada; 12 Patient Partner, 403 rue des Érables, Neuville, Québec G0A 2R0 Canada; 13 Healthwise, Incorporated, 40 Court St, Suite 300, Boston, MA 02108 USA; 14 Department of Family Medicine and Emergency Medicine, Faculty of Medicine, Laval University, 1050 avenue de la Médecine, Quebec, QC G1V 0A6 Canada; 15 Research Centre of the CHU de Québec, CHU de Québec, 10 de l’Espinay, Quebec, QC G1V 0A6 Canada; 16 School of Nursing and Ottawa Hospital Research Institute, University of Ottawa, 451 Smyth Road, Ottawa, ON K1H8M5 Canada; 17 Department of Health Services Research, The University of Texas MD Anderson Cancer Center, 1400 Pressler St., Houston, TX 77230 USA

**Keywords:** Patient engagement, Vulnerable populations, Marginalized populations, Decision aids, Shared decision making

## Abstract

**Background:**

Patient decision aids aim to present evidence relevant to a health decision in understandable ways to support patients through the process of making evidence-informed, values-congruent health decisions. It is recommended that, when developing these tools, teams involve people who may ultimately use them. However, there is little empirical evidence about how best to undertake this involvement, particularly for specific populations of users such as vulnerable populations.

**Methods:**

To describe and compare the development practices of research teams that did and did not specifically involve members of vulnerable populations in the development of patient decision aids, we conducted a secondary analysis of data from a systematic review about the development processes of patient decision aids. Then, to further explain our quantitative results, we conducted semi-structured telephone interviews with 10 teams: 6 that had specifically involved members of vulnerable populations and 4 that had not. Two independent analysts thematically coded transcribed interviews.

**Results:**

Out of a total of 187 decision aid development projects, 30 (16%) specifically involved members of vulnerable populations. The specific involvement of members of vulnerable populations in the development process was associated with conducting informal needs assessment activities (73% vs. 40%, OR 2.96, 95% CI 1.18–7.99, *P* = .02) and recruiting participants through community-based organizations (40% vs. 11%, OR 3.48, 95% CI 1.23–9.83, *P* = .02). In interviews, all developers highlighted the importance, value and challenges of involving potential users. Interviews with developers whose projects had involved members of vulnerable populations suggested that informal needs assessment activities served to center the decision aid around users’ needs, to better avoid stigma, and to ensure that the topic truly matters to the community. Partnering with community-based organizations may facilitate relationships of trust and may also provide a non-threatening and accessible location for research activities.

**Conclusions:**

There are a small number of key differences in the development processes for patient decision aids in which members of vulnerable populations were or were not specifically involved. Some of these practices may require additional time or resources. To address health inequities, researchers, communities and funders may need to increase awareness of these approaches and plan accordingly.

**Electronic supplementary material:**

The online version of this article (doi:10.1186/s12911-016-0399-8) contains supplementary material, which is available to authorized users.

## Background

Patient decision aids are a core tool of shared decision making. These tools, often in the form of booklets, websites, videos, or a combination of media, are intended to help people make health decisions by providing information about the potential benefits and harms of their options, along with support for determining how well or poorly each option aligns with what matters to the person or people most affected by the decision. As evidenced in a meta-analysis of 115 trials, these tools increase the likelihood of people making evidence-informed, values-congruent health decisions [1]. The International Patient Decision Aids Standards stipulate that patient decision aids should be developed with user involvement [2, 3], meaning patients, caregivers, families, surrogate decision makers and health care professionals, as appropriate. Involving users in the development of patient decision aids is recommended because, in principle, such involvement should help patient decision aids better meet the needs of the people for whom they are intended, rendering the tools more acceptable, comprehensible, useful, and usable. However, despite the general recommendation in favor of involving people in the development of patient decision aids that they or others like them may use, there is little guidance and scant evidence regarding optimal practices for such involvement.

The question of how best to involve people in the development of a patient decision aid may be particularly important when those people are members of vulnerable populations. A meta-analysis demonstrated that shared decision-making interventions, including but not limited to patient decision aids, may be more beneficial to some vulnerable populations, specifically, those with lower socioeconomic status and literacy [4]. Other work has similarly highlighted the potential of patient decision support tools for members of vulnerable populations [5, 6]. From a public health perspective, patient decision aids may therefore contribute to the overall objective of reducing health inequities. However, to achieve this objective, patient decision aids must be usable by members of vulnerable populations who already experience additional difficulties when engaging in shared decision making [7]. Furthermore, members of vulnerable populations are still under-represented in health research overall [8], and may also be less represented in the development of patient decision aids, resulting in tools that may be difficult for them to use [9]. Ensuring that members of vulnerable populations can participate in shared decision making is essential to avoid perpetuating—or worse, exacerbating—health inequities [10, 11]. Developing patient decision aids that work for members of vulnerable populations is part of this effort.

According to Flaskerud and Winslow’s conceptual framework, vulnerable populations are defined as social groups with a higher risk of health problems [12]. These groups include people who are poor, discriminated against, stigmatized, marginalized or disenfranchised [12]. People who are members of vulnerable populations may be disadvantaged, for example, due to psychological or cognitive characteristics (e.g., mental illness, low literacy), socio-economic or cultural characteristics (e.g., education, income, race, language) or may experience discrimination or stigma for other reasons (e.g., alcohol or drug dependencies, sexual orientation) [12, 13].

A systematic review on strategies for improving health and medical research involving socially disadvantaged groups [8] showed that to better represent members of vulnerable populations, research teams may have to anticipate higher budgets, longer timeframes and the need to establish partnerships with communities. However, it is unknown to what extent these findings apply to the development of tools such as patient decision aids.

To improve the knowledge and health care decision making of vulnerable populations, it is important to create patient decision aids that are meaningful, understandable, and usable for the people who will use them. Our study therefore aimed to describe how members of vulnerable populations have been specifically involved in the development of patient decision aids, and to investigate which, if any, development steps might be similar or different between development processes that did and did not specifically involve members of vulnerable populations.

## Methods

### Study design

Our study used a mixed methods design [14, 15] structured in two phases. In phase 1, we conducted a secondary analysis of a systematic review about how different teams have developed patient decision aids to quantitatively compare practices of teams that did and did not explicitly involve members of vulnerable populations. In phase 2, we conducted semi-structured interviews with 10 decision aid developers: 6 who involved members of vulnerable populations and 4 who did not. These interviews were designed to help us explore concepts identified in phase 1 in greater depth, along with other themes that were not possible to extract from published reports. Our study was approved by the Research Ethics Committee of Laval University (approval number 2014-035/26-05-2015).

### Phase 1

#### Data collection

Our systematic review methods are described in full elsewhere [16]. Briefly, we included articles that described at least one step of the development of a patient decision aid, with no other inclusion or exclusion criteria. Two independent analysts extracted data about development processes based on a conceptual framework of user-centered design [16]. We reconciled disagreements at regular meetings and contacted authors by email to validate extracted data. For this study, we used only data that we were able to validate with the original authors.

In order to achieve the aims of this study, we identified development processes specifically involving members of vulnerable populations. First, we developed a preliminary set of criteria to identify these projects when extracting data. The criteria were based on Flaskerud and Winslow’s framework [12] and the Centers for Disease Control and Prevention’s public health workbook on reaching at-risk populations in an emergency [13]. Two independent reviewers (MD, MET, both Master’s students in community health with backgrounds in sociology) then re-reviewed all extracted data along with the original article or articles for each project. These reviewers independently classified each development process into one of two groups: projects that specifically included members of vulnerable populations and projects that did not. These reviewers also classified the type of vulnerability into one or more of eight categories based the frameworks noted above: (a) race and ethnicity, (b) lower socioeconomic status (lower education or lower income), (c) lower literacy, (d) mental health problems, (e) physical disabilities, (f) older adults (65+), (g) children and adolescents (18-), (h) other. The reviewers met regularly to discuss and resolve any disagreements.

To validate data extracted from published articles, we contacted authors of papers included in our systematic review by email and asked them to verify the extracted data or to provide clarification, corrections or additional information as necessary. As part of this process, we specifically asked about the involvement of members of vulnerable populations during the development of their patient decision aid.

#### Analyses

To determine whether any differences exist in development practices between teams that did and did not involve members of vulnerable populations, we conducted quantitative analyses to explore which, if any, variables relevant to the development of a patient decision aid are significantly associated with the involvement of members of vulnerable populations. Our dependent variable was therefore whether or not the project specifically involved members of vulnerable populations. We identified 31 potential independent variables in our data matrix that were of primary interest to us due to their importance within our conceptual frameworks of user-centered design and vulnerability, and had acceptable distribution properties within the secondary data set. We present the full list of these variables along with summary statistics in Additional file 1: Appendix 3. We used bivariate analyses with a threshold of *P* > .20 to rule out any variables that were unlikely to be associated with our dependent variable and entered all remaining variables into a multivariable logistic regression.

To inform sampling plans in phase 2, for all independent variables in our regression, we also determined whether any differences existed between two groups of categories of vulnerability using Fisher Exact tests. The first group included the categories race and ethnicity, lower socioeconomic status, lower literacy, and mental health problems. The second group included physical disabilities, older adults, children and adolescents, and other categories of potential vulnerability. We performed all analyses in R, version 3.2.1 [17].

### Phase 2

#### Data collection

In order to explore the involvement of members of vulnerable populations in more depth, in phase 2 we conducted semi-structured telephone interviews with developers of patient decision aids. We elected to interview developers as these are the people who planned and conducted the development processes.

Recognizing that there are many ways in which vulnerability may present and to remain within the scope of our project, we elected to focus on the social and economic dimensions of vulnerability (race and ethnicity, lower socioeconomic status, lower literacy, mental health problems). We identified authors who had developed patient decision aids (hereafter referred to as developers) from the projects included in the systematic review in phase 1 using maximum variation sampling [18]. Specifically, we aimed to maximize diversity with respect to the types of vulnerable populations involved within our subgroup and the development methods used. To select projects from which we wished to interview developers, during data extraction, analysts (MD, MET, TP, GV, EB) indicated, according to their subjective assessment, any article that they believed described an especially high quality development process. We then pooled all articles that had been thus flagged. Two other team members (SCD, HW) independently screened these articles and came to consensus on which project teams might best be able to provide the widest range of insights, consulting with the lead author (MD) in cases of questions regarding vulnerability. When selecting projects for interviews, in addition to seeking maximum variation, we prioritized projects with more recently published articles to facilitate recall and to better capture current methods.

We contacted the corresponding authors of articles associated with each of these projects to invite them to participate in a 60-min telephone interview conducted by members of the research team (SCD for all interviews, MD for interviews with teams identified as having involved members of vulnerable populations). We offered authors an honorarium of CAD$100 in appreciation of their time. We aimed to interview ten teams approximately balanced between those that did and those that did not specifically involve members of vulnerable populations.

The interviews focused on six main themes: (1) Description of the development process, (2) Goals of the development process, (3) Role of patients in the project, (4) Nature and level of patient participation in the development process, (5) Barriers and facilitators to involving members of vulnerable populations, (6) Lessons learned. Additional file 1: Appendix 1 shows the interview guide. To glean insights from developers regarding differences in development practices that we identified in phase 1, we specifically asked developers who had involved members of vulnerable populations, “Your study was identified as one of the studies that included users who may be from socially or economically disadvantaged populations. In our quantitative analyses we found the following differences between studies that did and did not involve people from such populations: [describe differences]. I’m wondering if you can comment on those differences? To what extent do these findings reflect or fail to reflect your own experiences in this project?”

In addition to developers, we originally planned to also interview patients who had been involved in the development processes. However, numerous obstacles that we were unable to resolve (e.g., Institutional Review Board regulations, losses of contact information, principal investigators having changed institutions) prevented us from doing so.

#### Analyses

We transcribed interviews verbatim and two independent researchers (MD, MET) analyzed them qualitatively in NVivo 10, following standard steps of deductive thematic analysis [19]. The lead author (MD) generated a set of initial themes. Then, the lead and second author (MET) refined and sought themes in an iterative manner until we reached consensus and identified no new themes. We then reviewed, defined and named themes with two other authors (SCD, HW).

## Results

### Phase 1

Full results of the larger systematic review will be described in a companion paper (G. Vaisson, personal communication [20]). Briefly, we identified 443 publications about the development of a patient decision aid. After consolidating multiple articles from the same projects, our final data set consisted of 283 patient decision aid development projects. We were able to obtain confirmation or correction of our extracted data from 187 out of 283 teams, for a total response rate of 66%. Within the 187 projects, 30 (16%) specifically involved members of vulnerable populations in the development process. The full list of projects and articles is available in Additional file 1: Appendix 2.

Our multivariate logistic regression identified 2 out of 10 variables that were significantly associated with whether or not patient decision aid development processes specifically involved members of vulnerable populations. Conducting informal needs assessments and working with community-based organizations were associated with the specific involvement of members of vulnerable populations. Table 1 shows frequencies and regression results. (See Additional file 1: Appendix 3 for full details of all non-retained variables.)

**Table 1:** Associations between development process variables and involvement of members of vulnerable populations

Variable	Projects that specifically involved members of vulnerable populations (*n* = 30)	Projects that did not specifically involve members of vulnerable populations (*n* = 157)	Odds Ratio (95% Confidence Interval), *P* value
Decision aid users were involved in an informal needs assessment	22 (73%)	63 (40%)	2.96 (1.18,7.99), *P* = .02*
Decision aid users were involved in a content review prior to prototype development	9 (30%)	25 (16%)	0.96 (0.31,2.71), *P* = .94
Decision aid users were involved in a pilot test	26 (87%)	111 (71%)	1.75 (0.53,7.03), *P* = .37
Developers asked users their thoughts & opinions of the tool	27 (90%)	117 (75%)	1.67 (0.48,7.83), *P* = .44
Developers assessed the impact of the decision aid on users	25 (83%)	146 (93%)	0.49 (0.12,2.10), *P* = .32
An advisory panel of users was involved in the project	8 (27%)	16 (10%)	2.04 (0.61,6.47), *P* = .24
Decision aid users were recruited through community-based organizations	12 (40%)	17 (11%)	3.48 (1.23,9.83), *P* = .02*
Decision aid users were compensated/incentivized in some way	16 (53%)	54 (34%)	1.41 (0.55,3.54), *P* = .47
An expert panel of academics, clinicians, etc. was involved in the project	22 (73%)	90 (57%)	1.76 (0.66,5.10), *P* = .27
The project team had formal links with a specific patient or consumer organization	7 (23%)	21 (13%)	0.87 (0.23,2.92), *P* = .83

*P < .05

There were no significant differences between the categories of vulnerability in these 10 variables. This lack of difference supported our sampling plans for phase 2.

### Phase 2

We identified 14 projects for potential interviews. Out of these, 1 developer team did not respond to our request and 3 declined to participate due to time restrictions or other precluding conditions, including project leaders being away on leave. Thus, we interviewed ten developer teams in total (71% of those invited): 6 that had specifically involved people from populations that may be vulnerable due to race and ethnicity, lower socioeconomic status, lower literacy, or mental health conditions (projects numbered 1 to 6 in quotes), and 4 that did not (projects 7 to 10 in quotes) for comparison. One project had two team members participate; for all other projects we interviewed a single identified team leader.

In order to address our goal of unpacking differences identified in phase 1 about development practices of teams that involved members of vulnerable populations compared to those that did not, our analyses identified 5 themes that were specific to developers who involved members of vulnerable populations. We also identified 4 themes that were shared across all the interviews, meaning that some aspects of involving vulnerable populations were not different from involving members of other types of populations. Finally, we identified barriers and facilitators to involving members of vulnerable populations in the development of patient decision aids.

### Themes specific to involving members of vulnerable populations

We identified three themes that explained the greater likelihood of informal needs assessments in projects in which members of vulnerable populations were specifically involved. The informal activities served to center the decision aid around users’ needs, to better avoid stigma, and to ensure that the topic truly matters to the community. Two themes provided more insight into the greater likelihood of recruiting participants through community-based organizations. First, developers reported that it is critical to build relationships of trust with the community, a process that may be facilitated by the structure of an established community group. In addition, community-based organizations offer an important function by providing a nonthreatening and often more feasible location for project activities.

#### Centering the decision aid around users’ needs

One theme that emerged from developers who involved members of vulnerable populations is the importance of adapting the design to better address users’ needs and perspectives. They noted that this may be easier to do earlier in the development process and requires not being too fixated on the original plan.

“I think it would be really to keep an open mind and not go into a project thinking that you know exactly how it’s going to go and what the end result is going to be. Because, when you do that, you start to put aside what you’re [seeking] from your target audience. […] And to be as open minded as you can. What it’s going to look like at the end is really a key component to making sure that it ends up the way that your audience really wants it to be.” (Project 4)

#### Avoiding stigmatizing representation

Developers indicated that they paid attention to how to best represent members of vulnerable populations not only in the final product, but also in every step of their process. From the recruitment process to team interaction during development activities, developers who involved members of vulnerable populations worked with communities to ensure representations that avoided discrimination and stigma. They focused on labels, how to represent people, what wording to use and how to avoid potentially stigmatizing imagery or text, including in logos and video:

“… how to film people, how to present people, even down to the choice of who is going to be the narrator. And the importance of gender, and race, ethnicity…” (Project 1)

#### Choosing a topic that matters to the users’ community

For projects that specifically involved members of vulnerable populations, developers told us that they perceived users’ involvement as being motivated by their commitment to a topic that affected them and their community. Developers saw the people involved in the projects as wanting to make a positive difference for their community and that, by participating in the project, people were contributing to the community to which they belonged.

“I certainly can’t talk for them but I think that they felt it was important, they felt that it was something they were doing for their own community. And they could come share their experience in a way that would be helpful to their community. So yeah, I think that they felt that it was an important thing for them to do.” (Project 4)

“I think the topic helped, because it was something that there seemed to be a genuine interest in.” (Project 6)

#### Taking the time to build relationships of trust

When involving members of vulnerable populations, developers told us that it is important to take time to build trust-based relationships of mutual respect. Participants pointed to the importance of building trust-based relationships early in the project and often before the development of the tool itself. These relationships help ensure that people feel comfortable participating in project activities and that their participation is maintained throughout the process. Developers reported that it took time to build these relationships.

“I think there was one focus group that we had scheduled that I think maybe only two people showed up and so… We had to kind of reschedule that and… So that took a lot of time and patience on the part of our research staff to really try to build a relationship with some of these patients so that they were trustful.” (Project 4)

“It’s not something that you can just show up at the […] event … contacting them a week in advance and walking in and conduct a focus group… It takes a long time to develop those relationships and to build the trust that is needed in order to do these kinds of studies.” (Project 5)

#### Meeting people in their environment

Developers who specifically involved members of vulnerable populations emphasized the importance of a development process that is not medicalized. For this reason, their recruitment processes and project activities typically took place in community-based settings. Having researchers go to the community, rather than vice versa, facilitates both the recruitment process and ongoing user participation throughout the project. It may also help members of the community feel more comfortable and less stigmatized. On a more practical level, such logistical choices mean that people may also be less likely to face barriers to participation such as transportation time and costs.

“I think if you’re dealing with a clinically hard-to-reach population who don’t have access to additional services, then a community setting can offer that way in to people that are otherwise difficult to reach. […] I think what certainly did help was to use a community-based setting in our research because it was a friendly environment, it was a place where people were comfortable and happy going in where they felt supported and it was somewhere that didn’t feel particularly medicalized. So it was, it had kind of a casual environment to it, it was somewhere where people felt happy dropping in and staying for, you know, a cup of tea and a chat. So it did kind of put that sort of feeling around rather than feeling like you were being assessed clinically.” (Project 6)

### Themes shared across all the interviews

#### The importance of involving users in the process of developing a patient decision aid

A majority of developers emphasized that they felt it was important to involve patients in the development process because they are the ultimate users of the decision aid. Patients bring their own perspectives to the table that developers may not otherwise have, and can help ensure the tool ultimately reflects what matters to its users.

“We really do believe in stakeholder engagement. […] It really enhances the work that we do. […] I think what often happens is we don’t engage them. We know what the answer is, and we put something together and then we will engage them after the final steps, right? But, here, we’re engaging them at every step, to make sure that we’re on the right track. They’re really guiding us, you know?” (Project 1)

“But, we don’t generally involve the patients: what they want to know, how they understand things. So, I’d say that you need to start involving the end user in the process, you need to start thinking about that, because a lot of people don’t even do that.” (Project 8)

#### Patients’ feedback can lead to many changes to the content and the format of the tool

Developers told us that patients had significant impact on the decision aid. Major modifications were made to decision aids based on their feedback. Some developers even noted that they revamped the entire tool based on patients’ feedback. The majority of developers told us that by incorporating patients’ feedback, they felt that their decision aid changed for the better and that if they had to do it again, they would do the same.

“And I am so glad that we did that, because […] I think we started off with what we tend to do, which is: OK, here’s something we developed. And almost, you know, of course you don’t say it this way, but in a way, what probably comes off: ‘Oh, here’s something we developed, isn’t it beautiful? Approve it.’ And I love that we have a pretty strong and vocal community advisory panel who said: Wait a second, wait a second. This doesn’t even really… you know, make sense.” (Project 2)

“It appeared that they saw things very differently and they thought differently. So we actually changed some important things based on their views.” (Project 9)

#### Involving users is time– and resource-intensive, but is worth it

Developers indicated that the more patients and other stakeholders were involved, the more useful input developers could get. They agreed that engaging more people pays off in the end; however, they also noted that this takes time. Some of them stated that they had underestimated the time and budget required for their development process and that they have learned to plan better through experience.

“What I do differently is that, now that I’ve done this a few times, I know how to write this better in a grant application. And allow the amount of time and resources that are needed to do it well.” (Project 2)

“Gosh, I think probably the main lesson is that, from my perspective, recruitment, it generally takes longer than you anticipate that it’s going to take. […] [However] I think it was very useful to involve people in the two stages that we did.” (Project 8)

#### The challenge of incorporating opposing opinions

Seeking significant feedback from users may mean that developers encounter conflicting opinions held by different stakeholders. Developers found it challenging to reconcile these different opinions. As one developer said, “Some [patients] said we had too much, some said we had not enough,” (Project 10) challenging the development team to integrate all patients’ views.

### Barriers and facilitators to involving members of vulnerable populations

We explicitly asked developers who had specifically involved members of vulnerable populations in the development process of patient decision aids about the barriers and facilitators of such involvement, summarized in Table 2. We note that many facilitators were responses to barriers.

**Table 2:** Barriers and facilitators to involving people who are members of vulnerable populations

Barriers	
1. **Scheduling:** People are busy, may be sick, and research teams have to consider multiple and possibly conflicting schedules to be able to gather everyone together. 2. **Transportation:** Transportation costs can be a barrier to members of vulnerable populations. 3. **Ethical procedures:** Institutional review boards’ established procedures may not be suited to some populations; for example, detailed consent forms may present difficulties for people with lower literacy, even when read aloud. 4. **Lack of trust:** People may refuse to participate due to a lack of trust in the research team. 5. **Finding an appropriate workload**: It can be difficult to find the sweet spot between enough involvement for meaningful participation but not so much that it becomes overwhelming. 6. **Project planning**: Projects that involve members of vulnerable populations may require more time, possibly a bigger budget and more planning, which may or may not be feasible within the constraints of funded research projects.	
Facilitators	
1. **Flexibility in scheduling**: Teams should work around scheduling constraints, including people’s other commitments such as work and caregiving activities. 2. **Location**: A community-based setting helps reduce potential power imbalances and can also help with logistics. 3. **Favourable institutional environment:** It is helpful to work with an institutional review board or research ethics committee who already have knowledge or who are open to learning more about norms and best practices in research involving members of vulnerable populations. 4. **Relationship of trust**: The research team needs to take the time to build trust with patient partners from vulnerable populations and also with all the other people involved in the project, including community workers and health care professionals. 5. **Enjoyable methods**: Having activities that people enjoy can stimulate sustainable participation in project; for example, people may enjoy focus groups more than filling out questionnaires. 6. **Adapting the technology**: It may be necessary to adapt technology; for example, particularly when working with people who are members of populations with lower literacy and less access to internet, communication by email may not be ideal. 7. **Financial and material incentives**: Remuneration, honoraria, or material incentives provide a way to say thank you and to demonstrate the value and importance of people’s participation in the project. 8. **Relevance and importance of topic to the community:** Ensuring the topic is relevant and important to the community encourages interest and commitment.	

## Discussion

This study aimed to describe and compare the development practices of research teams that did and did not specifically involve members of vulnerable populations in the development of patient decision aids. Our study had four principal findings.

First, in terms of how they involved users in the development of a patient decision aid, there appeared to be relatively few differences between teams that did and did not specifically involve members of vulnerable populations. Even in an exploratory analysis of 31 potential variables describing development practices and surrounding issues, we found only 2 variables that differed significantly. Similarly, interviews revealed common themes about the importance of involving users, the value of involving them early and throughout the development process, and the need to allocate resources—both time and funds—to such involvement.

Second, the few differences that we did observe had to do with ensuring that the decision aid and its development process were centered around users and their communities. Such an approach may be either more important or simply more common when involving members of vulnerable populations. Results from our secondary analysis of systematic review data suggested that, when involving members of vulnerable populations, decision aid development processes were more likely to include an informal needs assessment, meaning any activities that help the team identify what matters to the people for whom the decision aid is designed. Such activities explicitly help center the decision aid around users’ needs. Our qualitative analyses unpacked this finding and suggested that this work may consist primarily of identifying what matters to the community and ensuring that materials do not evoke or perpetuate stigma and discrimination. In so doing, developers may encourage involvement that is rooted in people’s commitment to addressing a topic that matters to their community. In this sense, these activities may help enact or reinforce community agency and power by explicitly providing community members an opportunity to express what matters to them and to their community.

Third, building trust was a theme that stood out in every interview with developers who involved members of vulnerable populations in their project and may be another aspect of informal needs assessments. Trust-based relationships played a role in the recruitment process and in maintaining users’ participation throughout the entire development process. Recruiting members of vulnerable populations to a research project is facilitated when a relationship of trust is established before beginning the project. Interviewees reported that this often meant that the principal investigator or other team members already had a link with a particular community. In some cases, team members were also members of the population in question, and thus served in a bridging role. Established relationships may help to ensure better representation of community needs within a project and also help to avoid what is sometimes called parachute research, meaning when researchers come into a community, collect the data they need, and never return.

Fourth, related to our third point, our quantitative analyses also suggested that specifically involving members of vulnerable populations is associated with collaborating with a community-based organization for recruitment. Qualitative analyses further suggested that teams not only recruit through community-based organizations, but also do the work there. When development activities and other aspects of the research project take place in community-based settings, it can reduce logistical barriers and may also reduce power imbalances.

### Limitations

Our findings present four main limitations. First, due to a number of barriers, we were not able to interview patients who had been involved in the development processes. We believe developers provided credible and valuable insights to understand differences in development practices when involving vulnerable populations; however, to fully understand the processes, it would have been preferable to interview some of the people they involved as well. Second, because our data came from a systematic review, we were limited by what was reported in publications. All of the data we used in this study were validated by the original authors; however, there may be important aspects of the development process that we were unable to capture; for example, the friendliness and openness of the team members. Third, for phase 2 of our study, we focused on specific categories of potential vulnerability: people who may be marginalized, face discrimination or face stigma due to lower education, income or literacy, race, ethnicity, or mental health conditions. We made this choice for reason of scope, and these categories of vulnerability were more highly represented in our sample. However, it is possible that this choice may mean that our results do not apply to other reasons for which a population might be vulnerable. Fourth, the question of how to best involve the people who may ultimately benefit from research—patients and other stakeholders—is not exclusive to patient decision aid research. Our work focused on patient decision aid development because it provided a rich literature of descriptions of development processes and because questions of how best to involve users are of primary interest when they will be directly using the products of the project. However, this choice of focus may mean that our findings may not be applicable outside the sphere of patient decision support and shared decision-making research.

### Comparison with prior work

Bonevski and colleagues [8] found several barriers and facilitators to the involvement of members of socioeconomically disadvantaged groups in medical and health research. Their review addressed research in general, which includes not only development of patient-oriented tools like decision aids but also randomized controlled trials, observational cohort studies, and other research methods. Their analysis examined barriers at five stages of a typical study: sampling, recruitment, data collection, intervention delivery and retention throughout the study, if applicable, and they reported a wide array of barriers across these five stages, including lack of trust, fear of being publicly exposed and concern that the research may cause harm or stigma. As with our findings, partnering with community groups was a commonly-used approach in the included studies to address such barriers, and the authors also synthesized a number of other facilitators including avoiding “fly in, fly out” research that fails to give back to the community, adapting study materials in terms of literacy levels or technology use, and various approaches to ensuring cultural competence.

Our findings can also be placed in the context of a long history of participatory action research in health. Participatory action research emphasizes the importance of involving communities in the research process, aims to avoid power imbalances and build trust, and prioritizes working with a community to effect change [21, 22]. These three principles can be seen in our findings. All developers we interviewed emphasized the importance of working with research end users, but those who involved members of vulnerable populations spoke specifically about involving communities. The second principle is reflected in themes we identified around researchers going to the community, paying close attention to issues of stigma and building relationships of trust. However, our comparison with this principle is challenged by the fact that we were unable to interview any of the patients who had been involved in these projects. This may be a function of the overall research enterprise and the way ethical oversight works within it, as well as our decision to approach teams by way of published articles. Had we sought out projects by directly contacting community-based organizations, we might have had different results. The third principle of participatory action research, effecting change, is less well reflected in our findings. However, in the case of patient decision aids, if the decision support is needed within a community, developing a patient decision aid is one way to help bring about that change.

Our work is also situated in the context of a rapidly evolving literature on patient, public and service user involvement in research projects of various kinds. Many contributions to this literature have highlighted similar issues to those identified in our study, including more time and funding required [23, 24] and the importance of working in partnership with communities [25, 26] including going to the community and conducting activities in the community’s environment [27].

## Conclusions

Developers of patient decision aids who do or do not specifically involve members of vulnerable populations have similar development practices overall, with a few key differences. Those who involve members of vulnerable populations may be more likely put more focus on centering the decision aids around its eventual users by conducting activities to assess their needs and by pilot-testing the decision aid with potential users. They are also more likely to recruit participants through community-based organizations. These practices require partnering with communities before and throughout a project to build and maintain relationships of trust.

These approaches are likely to have benefits for working with any population or community, but may be particularly important when working with members of vulnerable populations, who stand to benefit the most from these tools. It is important to ensure that patient decision aids are usable by all so that these tools can be better used in real-world settings. However, identified facilitators for involving members of vulnerable populations may require time and resources that go beyond a typical funded research project. Funders could help address this by explicitly allowing longer lead-up times to funding application deadlines or by enabling the early stages of projects to focus on needs assessment and relationship-building between researchers and communities. Decision aid developers in our study often had existing relationships with communities that they had built over time. Although it may be difficult to assess, funders may wish to consider funding criteria that includes the strength and sustainability of such existing relationships. Researchers, communities and funders could work together to ensure that the needs and perspectives of members of vulnerable populations are incorporated into project planning. Finally, funders may wish to initiate, continue or increase support for community-initiated or community-driven research.

## Additional file

Additional file 1: Appendix 1. Interview guide. **Appendix 2.** Included projects. **Appendix 3.** Variables considered. (DOCX 390 kb)

## Abbreviations

CI: Confidence interval
OR: Odds ratio
