# Supplementary material for: Involving members of vulnerable populations in the development of patient decision aids: a mixed methods sequential explanatory study
Source: BMC Med Inform Decis Mak. 2017 Jan 19;17:12. doi: 10.1186/s12911-016-0399-8 (PMC5244537; doi:10.1186/s12911-016-0399-8)
Supplement: Additional file 1: Appendix 1. — Interview guide. Appendix 2. Included projects. Appendix 3. Variables considered. (DOCX 390 kb) [file 12911_2016_399_MOESM1_ESM.docx]

**Appendix 1. Interview Guide**

**PCORI Interview Protocol**

**Developers/Researchers**

***Interview objectives***

1. To explore the perceptions and experiences of people who have previously developed decision aids and have involved potential users (patients, clinicians, others) in diverse ways.
2. To develop ideas of how to improve development processes.

***Procedure***

- Connect and start the recorder
- Introduce yourself: “Hello, this is [name] from Laval University in Quebec City, Canada. I’m calling you as we had agreed. Is this still a good time for us to talk?”
- Remind person of goal of study: “Thank you for your willingness to talk to us today. As we said in our email/letter to you, the goal of this interview is to find out more about your experience in the project you led/participated in with [name of contact] in [year] about [topic, e.g., development of name of decision aid]. There are no right or wrong answers to any of these questions. We hope that you will be candid and honest with your responses, we would really like to learn from you and your experience. Does this sound OK so far to you?”
- Obtain participants’ informed consent. Key points:
- “As you know, this is a research study. We’ve already emailed you a copy of the full consent form, the questions we will be asking you, and what we understand about your development process from your papers. This conversation will take about an hour. If there are any questions that you don’t want to answer, you don’t have to answer them. If you need to leave for any reason, just let me know and I will stop. If we stop early, you can decide whether it’s ok if we keep the data we’ve already collected, or if you’d rather we discard it. Everything you say is confidential. We won’t be identifying particular projects in our reports. Is this OK with you?”
- “We are going to be audio recording this phone call, then transcribing it verbatim, taking care to remove any identifying information at that stage. After transcription, we will destroy the recording. Is it OK with you if we record this call?”
- “We will keep a separate file of your name and contact information for 7 years. We will keep this in a locked file cabinet in our offices at the university. Is that OK with you?”
- “Just a reminder that we would like to compensate you for your time. At the end of the interview, we will take your mailing address to send you a check. If you have to stop the interview early, we can take your address then.”
- **Do you have any questions or comments for me before we get started?**
- Conduct the interview
- Collect mailing address, record in a separate file to send compensation.

**START OF THE INTERVIEW**

1. To begin with, please tell me about yourself/your team and the impetus for [name of project/decision aid]. Why did you create this decision aid?
2. Tell me a little about the approach you used in the development of [name of decision aid]:

**QUESTIONS (make sure to ask each item not covered spontaneously)**

- Underlying model or framework that guided the process
- Mix of people, who was involved
- Process used
- Financial or material resources to support the process
- About how long did the development process take?

1. We’d like to talk a little about potential users involved in the process. When I say ‘users’ I mean people like patients who might someday use the decision aid to help them make a decision, or clinicians like physicians or nurse practitioners who might someday offer or recommend the decision aid to their patients. Can you tell me a little about the selection and participation of users in the development process?

**QUESTIONS (make sure to ask each item not covered spontaneously)**

- Who was involved
- How were they identified or selected?
- Were patients involved? If so, how?
- DEPENDING ON DECISION also ask about caregivers, surrogates, families.
- Were clinicians who are not decision aid researchers involved? If so, how?
- Was this the first time you involved users in this way?

[If needed, draw on extracted data to help inform this question]

1. Now if we think specifically about the users who were involved, we’d like to talk a little more specifically about their involvement.

**QUESTIONS (Make sure each one is addressed)**

- First of all, do you feel like you had a group of users that provided all the needed perspectives? Do you feel like there were any particular perspectives that were missing from the process? What made it easy or hard to get the particular perspectives that you needed?
- What do you see as the main **barriers** to user participation in the development process? (Prompts: recruitment, funding, institutional review boards, etc. Feel free to bring up things previous interviewees have said.)
- What do you see as the main **facilitators** to user participation in the development process? (Prompts: patient advisory boards, funders that require it, patient support groups, etc. Feel free to bring up things previous interviewees have said.)
- Was users’ feedback incorporated into your decision aid? If yes, how did you incorporate it? If no, what made it hard to incorporate?

1. [if identified as vulnerable populations] Your study was identified as one of the studies that included users who may be from socially or economically disadvantaged populations. In our quantitative analyses we found the following differences between studies that did and did not involve people from such populations: [describe differences]. I’m wondering if you can comment on those differences? To what extent do these findings reflect or fail to reflect your own experiences in this project?
2. [if users involved] Was there any sort of preparation or training for you or for the rest of the team to help everyone work and communicate with users?

**PROMPTS**

- If yes, please explain
- If no, do you wish there had been?

1. Thinking back to the way the project unfolded, what made it hard or easy to have patients and other potential users involved?

**PROMPTS**

- What, if anything, made it hard to havethem involved in the way they were?
- What, if anything, made it easy to havethem involved in the way they were?

1. Do you feel that user participation had an impact:

**QUESTIONS (Make sure each item is covered. Ask if not covered spontaneously.)**

- On the process?
- On the decision aid that was produced [name of decision aid if available]?
- On how much the decision aid did or did not help the target population?
- On their experience as a patient/caregiver/surrogate/person who might someday make this decision?
- On the attitudes of the other people involved?
- Directly or just by being the room?
- On your experience as a developer/researcher?

1. Today, what are your feelings about involving users in the development of tools like [name of decision aid]?

**QUESTIONS (make sure to ask each item not covered spontaneously)**

- Would you do it again the same way?
  - - PROMPTS:
      - What would you keep the same?
      - What would you do differently?

1. Lessons learned “if you were talking to a group of colleagues doing this kind of work, what would you advise and why?”

**QUESTIONS (make sure to ask each item not covered spontaneously)**

- Identification of users
- Recruitment of users
- Numbers and mix of people
- Preparation or training
- Involvement (type, level and timing)
- The particular experience or perspective that users bring
- Incentives or other supports to participation

1. Do you have any other thoughts that you would like to share?

Thank you so very much for making the time to talk to me.

**Appendix 2. Included Projects**

| **Article(s)** | **Did the project specifically involve members of vulnerable populations?** | **Reason(s) people included or involved may be vulnerable** | **Clinical context of patient decision aid** |
| --- | --- | --- | --- |
| Allen et al., 2009[28] | Yes | Race and Ethnicity | Prostate Cancer |
| Anderson et al., 2011[29] | No (but they were included within a diverse population) | Older Age | Ovarian Cancer |
| Arterburn et al., 2011[30] | Yes | Other: Obesity | Obesity |
| Au et al., 2011[31], Lam et al., 2013[32] | No (but they were included within a diverse population) | Lower Literacy | Breast Cancer |
| Auvinen et al., 2004[33] | No members of vulnerable population(s) reported |  | Prostate Cancer |
| Bailey Jr. et al., 2012[34], Bailey Jr. et al., 2013[35] | No (but they were included within a diverse population) | Race and Ethnicity; Lower Education or Income | Fragile X Syndrome |
| Barnieh et al., 2011[36] | No members of vulnerable population(s) reported |  | End-Stage Renal Disease |
| Barry et al., 1995[37], Barry et al., 1997[38], Piercy et al., 1999[39], Rovner et al., 2004[40], Holmes-Rovner et al., 2006[41], Wills et al., 2006[42], Pylar et al., 2007[43] | No (but they were included within a diverse population) | Older Age; Lower Education or Income | Benign Prostatic Hyperplasia |
| Bass et al., 2013[44] | Yes | Race and Ethnicity; Lower Education or Income; Lower Literacy | Colorectal Cancer |
| Bastian et al., 2002[45], McBride et al., 2002[46] | No members of vulnerable population(s) reported |  | Reproductive Health; Breast Cancer |
| Becker et al., 2009[47] | Yes | Other: Mobility Impairments | Reproductive Health |
| Bekker et al., 1999[48], Bekker et al., 2004[49] | No members of vulnerable population(s) reported |  | Reproductive Health; Down Syndrome |
| Berman et al., 2011[50] | No (but they were included within a diverse population) | Older Age | Abdominal Aortic Aneurysm |
| Berry et al., 2010[51], Berry et al., 2013[52] | No (but they were included within a diverse population) | Race and Ethnicity | Prostate Cancer |
| Breslin et al., 2008[53], Mullan et al., 2009[54] | No (but they were included within a diverse population) | Older Age | Diabetes |
| Brink et al., 2000[55], Fagerlin et al., 2004[56] | No members of vulnerable population(s) reported |  | Prostate Cancer |
| Brohan et al., 2014a[57], Brohan et al., 2014b[58] | Yes | Mental Health Condition(s) | Mental Health |
| Chewning et al., 1999[59] | Yes | Younger Age; Race and Ethnicity; Lower Education or Income; Lower Literacy | Reproductive Health |
| Chiew et al., 2008[60] | No (but they were included within a diverse population) | Older Age; Race and Ethnicity; Lower Education or Income | Breast Cancer |
| Consoli et al., 1995[61] | No members of vulnerable population(s) reported |  | Cardiovascular Health (Heart Disease, Stroke, etc…) |
| Costanza et al., 2011[62] | No (but they were included within a diverse population) | Race and Ethnicity; Lower Literacy | Prostate Cancer |
| Cox et al., 2012[63] | No (but they were included within a diverse population) | Race and Ethnicity | Prolonged Mechanical Ventilation |
| Dales et al., 1999[64], Wilson et al., 2005[65] | No (but they were included within a diverse population) | Older Age; Lower Education or Income | Advance Care Planning; Chronic Obsructive Pulmonary Disorder (COPD) |
| Davis et al., 1998[66] | No (but they were included within a diverse population) | Race and Ethnicity; Lower Education or Income | Vaccine Preventable Disease |
| Dillard et al., 2010[67] | No members of vulnerable population(s) reported |  | Colorectal Cancer |
| Dodin et al., 2001[68] | No (but they were included within a diverse population) | Older Age; Lower Education or Income | Reproductive Health |
| Dolan & Frisina, 2002[69] | No members of vulnerable population(s) reported |  | Colorectal Cancer |
| Dolan et al., 2013[70] | No (but they were included within a diverse population) | Race and Ethnicity | Knee Osteoarthritis |
| Dowding et al., 2004[71], Thomson et al., 2006[72] | No (but they were included within a diverse population) | Older Age; Other: Computer Literacy | Benign Prostatic Hyperplasia; Cardiovascular Health (Heart Disease, Stroke, etc…) |
| Drake et al., 2010[73] | Yes | Race and Ethnicity | Prostate Cancer |
| Durand, 2009[74], Durand et al., 2010[75], Durand et al., 2012[76] | Yes | Race and Ethnicity | Reproductive Health |
| Elit et al., 1996[77] | No members of vulnerable population(s) reported |  | Ovarian Cancer |
| Emmett et al., 2007[78], Montgomery et al., 2007[79], Frost et al., 2009[80], Hollinghurst et al., 2010[81] | Yes |  | Reproductive Health |
| Evans et al., 2007[82], Evans et al., 2010[83], Joseph-Williams et al., 2010[84] | No (but they were included within a diverse population) | Older Age | Prostate Cancer |
| Fagerlin et al., 2010[85], Fagerlin et al., 2011[86], Banegas et al., 2013[87], Korfage et al., 2013[88] | No members of vulnerable population(s) reported |  | Breast Cancer |
| Farnworth et al., 2008[89] | No members of vulnerable population(s) reported |  | Reproductive Health |
| Feldman-Stewart et al., 2000[90], Feldman-Stewart et al., 2001a[91], Feldman-Stewart et al., 2001b[92], Feldman-Stewart et al., 2002[93], Feldman-Stewart et al., 2004[94], Feldman-Stewart et al., 2006a[95], Feldman-Stewart et al., 2006b[96], Feldman-Stewart et al., 2012[97] | No (but they were included within a diverse population) | Older Age; Lower Education or Income | Prostate Cancer |
| Fetters et al., 2004[98], Ruffin et al., 2007[99], Taylor et al., 2010[101] | No members of vulnerable population(s) reported |  | Colorectal Cancer |
| Fiset et al., 2000[102] | No (but they were included within a diverse population) | Older Age; Lower Education or Income | Lung Cancer |
| Fleisher et al., 2008[103], Meropol et al., 2013[104] | No members of vulnerable population(s) reported |  | Cancer |
| Flood et al., 1996[105], Partin et al., 2004[106], Partin et al., 2006[107] | No (but they were included within a diverse population) | Older Age; Lower Education or Income; Lower Literacy | Prostate Cancer |
| Flood et al., 1996[105], Partin et al., 2004[106], Partin et al., 2006[107] | No (but they were included within a diverse population) | Older Age; Lower Education or Income; Lower Literacy | Prostate Cancer |
| Fortin et al., 2001[108], Bond et al., 2002[109], Col et al., 2007[110] | No members of vulnerable population(s) reported |  | Reproductive Health |
| Fraenkel et al., 2011[111], Fraenkel et al., 2012b[112] | No (but they were included within a diverse population) | Older Age | Cardiovascular Health (Heart Disease, Stroke, etc…) |
| Fraenkel et al., 2012a[113], Fraenkel et al., 2015[114] | No members of vulnerable population(s) reported |  | Rheumatoid Arthritis |
| Frank et al., 2010[115] | No (but they were included within a diverse population) | Older Age | Hospitalized patients (50% risk of death) |
| French et al., 2014[116] | No (but they were included within a diverse population) | Younger Age; Race and Ethnicity; Lower Education or Income | Reproductive Health |
| Frosch et al., 2001[117], Frosch et al., 2003[118] | No members of vulnerable population(s) reported |  | Prostate Cancer |
| Frosch et al., 2008a[119], Bhatnagar et al., 2009[120] | No members of vulnerable population(s) reported |  | Prostate Cancer |
| Frosch et al., 2008b[121] | Yes | Race and Ethnicity | Prostate Cancer; Colorectal Cancer |
| Gallo et al., 2010[122], Gallo et al., 2013[123], Wilkie et al., 2013[124] | No members of vulnerable population(s) reported |  | Reproductive Health; Sickle Cell Disease; Sickle Cell Trait |
| Garvelink et al., 2013[125] | No (but they were included within a diverse population) | Lower Education or Income | Reproductive Health; Breast Cancer |
| Green & Fost, 1977[126], Lerman et al., 1997[127], Green et al., 2001[128], Schwartz et al., 2001[129], Green et al., 2004[130], Green et al., 2005[131] | No (but they were included within a diverse population) | Race and Ethnicity; Lower Education or Income | Breast Cancer |
| Green & Levi, 2009[132], Hossler et al., 2011[133], Markham et al., 2015[134] | No (but they were included within a diverse population) | Older Age; Race and Ethnicity; Lower Education or Income; Mental Health Condition(s) | Advance Care Planning; Amyotrophic Lateral Sclerosis (ALS) |
| Gustafson et al., 1994a[135], Gustafson et al., 2001[136], Wise et al., 2008[137] | No members of vulnerable population(s) reported |  | Breast Cancer |
| Hamann et al., 2006[138], Hamann et al., 2007[139] | Yes | Mental Health Condition(s) | Schizophrenia |
| Hawkins Virani et al., 2013[140] | No members of vulnerable population(s) reported |  | Huntington's disease |
| Heinrich et al., 2012[141] | No members of vulnerable population(s) reported |  | Diabetes |
| Henderson et al., 2013[142] | Yes | Race and Ethnicity | Diabetes |
| Hightow-Weidman et al., 2011[143], Muessig et al., 2013[144], Muessig et al., 2014[145], Hightow-Weidman et al., 2015[146] | Yes | Race and Ethnicity; Lower Education or Income; Mental Health Condition(s); Sexual Orientation | Sexually Transmitted Infections (STIs); HIV |
| Hill-Briggs et al., 2008[147], Schumann et al., 2011[148], Majid et al., 2012[149] | No (but they were included within a diverse population) | Older Age; Race and Ethnicity | Diabetes |
| Hoffner et al., 2012[150] | No members of vulnerable population(s) reported |  | Cancer |
| Holbrook et al., 2007[151] | No members of vulnerable population(s) reported |  | Overall health; Cardiovascular Health (Heart Disease, Stroke, etc…) |
| Hollen et al., 2013a[152] | Yes | Younger Age; Cognitive Impairments | Substance Use |
| Hollen et al., 2013b[153] | No members of vulnerable population(s) reported |  | Prostate Cancer; Breast Cancer; Lung cancer |
| Holmes-Rovner et al., 2005[154] | Yes | Lower Literacy | Prostate Cancer |
| Hong et al., 2013[155] | No members of vulnerable population(s) reported |  | Cardiovascular Health (Heart Disease, Stroke, etc…) |
| Hooker et al., 2011[156] | No members of vulnerable population(s) reported |  | Breast Cancer |
| Hope & Rombauts, 2010[157] | No members of vulnerable population(s) reported |  | Reproductive Health |
| Hutchison & Campbell, 2002[158] | No members of vulnerable population(s) reported |  | Cancer |
| Ickenroth et al., 2014[159], Ronda et al., 2014[160] | No members of vulnerable population(s) reported |  | Diabetes; Cardiovascular Health (Heart Disease, Stroke, etc…) |
| Irwin et al., 1999[161] | No (but they were included within a diverse population) | Lower Education or Income | Breast Cancer |
| Jackson et al., 2010[162], Shourie et al., 2013[163], Tubeuf et al., 2014[164] | No members of vulnerable population(s) reported |  | Vaccine Preventable Disease |
| Jenkinson et al., 1998[165] | No (but they were included within a diverse population) | Older Age; Sexual Orientation | Prostate Cancer |
| Jibaja-Weiss et al., 2006a[166], Jibaja-Weiss et al., 2006b[167], Jibaja-Weiss et al., 2011[168] | Yes | Lower Literacy | Breast Cancer |
| Johnson et al., 2006[169] | No members of vulnerable population(s) reported |  | Endodontics |
| Johnson et al., 2010[170], Langston et al., 2010[171] | No members of vulnerable population(s) reported |  | Reproductive Health |
| Juan et al., 2008[172] | No members of vulnerable population(s) reported |  | Breast Cancer; Ovarian Cancer |
| Juraskova et al., 2008[173], Juraskova et al., 2014[174] | No (but they were included within a diverse population) | Older Age | Breast Cancer |
| Kellar et al., 2008[175], Mann E. et al., 2010[176], Marteau et al., 2010[177], Kellar et al., 2011[178] | No members of vulnerable population(s) reported |  | Diabetes |
| Kennedy et al., 2002[179], Kennedy et al., 2003[180] | No members of vulnerable population(s) reported |  | Reproductive Health |
| Kiatpongsan et al., 2013[181] | No members of vulnerable population(s) reported |  | Reproductive Health |
| Knapp et al., 2010[182] | No (but they were included within a diverse population) | Older Age | Cardiovascular Health (Heart Disease, Stroke, etc…) |
| Krist et al., 2007[183] | No members of vulnerable population(s) reported |  | Prostate Cancer |
| Krones et al., 2010[184] | No members of vulnerable population(s) reported |  | Cardiovascular Health (Heart Disease, Stroke, etc…) |
| Kuppermann et al., 2014[185], Norton et al., 2014[186] | No (but they were included within a diverse population) | Younger Age; Race and Ethnicity; Lower Literacy; Numeracy | Prenatal Care/Testing |
| Labrecque et al., 2010[187] | No members of vulnerable population(s) reported |  | Reproductive Health |
| Lalonde et al., 2004[188], Lalonde et al., 2006[189] | No (but they were included within a diverse population) | Obesity | Cardiovascular Health (Heart Disease, Stroke, etc…) |
| LaVista et al., 2009[190] | No members of vulnerable population(s) reported |  | Sickle Cell Disease |
| Lawrence et al., 2000[191] | Yes | Race and Ethnicity; Lower Education or Income; Lower Literacy; Numeracy | Breast Cancer |
| Légaré et al., 2007[192], Légaré et al., 2008[193], Menard et al., 2010[194] | No (but they were included within a diverse population) | Lower Education or Income | Reproductive Health |
| Légaré et al., 2011[195] | No members of vulnerable population(s) reported |  | Acute Respiratory Infections |
| Lerman et al., 1995[196] | No members of vulnerable population(s) reported |  | Breast Cancer |
| Lewis et al., 2010a[197], Miller et al., 2011[198], Pignone et al., 2011[199] | No (but they were included within a diverse population) | Older Age; Lower Education or Income; Lower Literacy | Colorectal Cancer |
| Li et al., 2013[200], Li et al., 2014[201] | No members of vulnerable population(s) reported |  | Rheumatoid Arthritis |
| Liao et al., 1996[202], Morgan et al., 2000[203] | No members of vulnerable population(s) reported |  | Cardiovascular Health (Heart Disease, Stroke, etc…) |
| Loh et al., 2007[204] | No members of vulnerable population(s) reported |  | Depression |
| Lurie et al., 2011[205] | No members of vulnerable population(s) reported |  | Herniated Disc |
| Mancini et al., 2006[206] | No members of vulnerable population(s) reported |  | Breast Cancer; Ovarian Cancer |
| Man-Son-Hing et al., 1999[207], Man-Son-Hing et al., 2000[208], McAlister et al., 2005[209] | No members of vulnerable population(s) reported |  | Cardiovascular Health (Heart Disease, Stroke, etc…) |
| Mathers et al., 2012[210], Ng et al., 2014[211] | No (but they were included within a diverse population) | Older Age; Lower Education or Income | Diabetes |
| Mathieu et al., 2007[212] | Yes | Older Age; Lower Education or Income | Breast Cancer |
| Mathieu et al., 2010[213] | No members of vulnerable population(s) reported |  | Breast Cancer |
| Matlock et al., 2014[214] | No members of vulnerable population(s) reported |  | Advance Care Planning |
| Mayer et al., 2010[215] | No members of vulnerable population(s) reported |  | Hematopoietic Stem Cell Transplant |
| McCaffery & Irwig, 2005[216], McCaffery et al., 2006[217], McCaffery et al., 2008[218], McCaffery et al., 2010[219] | No members of vulnerable population(s) reported |  | Reproductive Health; Cervical Cancer |
| McKay et al., 2005[220] | No members of vulnerable population(s) reported |  | Breast Cancer |
| Miller et al., 2005[221] | No members of vulnerable population(s) reported |  | Breast Cancer; Ovarian Cancer |
| Milne et al., 2009[222] | No members of vulnerable population(s) reported |  | Reproductive Health |
| Mitchell et al., 2001[223], Hanson et al., 2011[224], Snyder et al., 2013[225] | Yes | Lower Education or Income; Mental Health Condition(s) | Dementia |
| Montgomery et al., 2003[226], Emmett et al., 2005[227] | No members of vulnerable population(s) reported |  | Cardiovascular Health (Heart Disease, Stroke, etc…) |
| Montori et al., 2007[228], Weymiller et al., 2007[229], Jones et al., 2009[230], Nannenga et al., 2009[231], Mann D.M. et al., 2010[232] | No members of vulnerable population(s) reported |  | Diabetes; Cardiovascular Health (Heart Disease, Stroke, etc…) |
| Murray et al., 2001a[233] | No members of vulnerable population(s) reported |  | Benign Prostatic Hyperplasia |
| Murray et al., 2001b[234] | No members of vulnerable population(s) reported |  | Reproductive Health |
| Nassar et al., 2006[235], Nassar et al., 2007[236] | No members of vulnerable population(s) reported |  | Reproductive Health |
| Nozaki et al., 2007[237] | No members of vulnerable population(s) reported |  | Unruptured Cerebral Aneuryms |
| O’Connor et al., 1998a[238], O’Connor et al., 1998b[239], Rostom et al., 2002[240] | No (but they were included within a diverse population) | Older Age; Lower Education or Income | Reproductive Health |
| Onel et al., 1998[241] | No members of vulnerable population(s) reported |  | Prostate Cancer |
| Ozanne et al., 2007[242] | No members of vulnerable population(s) reported |  | Breast Cancer |
| Peate et al., 2011a[243], Peate et al., 2011b[244], Peate et al., 2012[245] | No members of vulnerable population(s) reported |  | Reproductive Health; Breast Cancer |
| Pencille et al., 2009[246], Montori et al., 2011[247] | No members of vulnerable population(s) reported |  | Osteoporosis |
| Perestelo-Perez et al., 2010[248] | No (but they were included within a diverse population) | Lower Education or Income | Benign Prostatic Hyperplasia |
| Perestelo-Perez et al., 2010[248] | No (but they were included within a diverse population) | Lower Education or Income | Depression |
| Perestelo-Perez et al., 2010[248] | No (but they were included within a diverse population) | Lower Education or Income | Hip or Knee Osteoarthritis |
| Permuth-Wey et al., 2010[249] | Yes | Race and Ethnicity | Breast Cancer; Ovarian Cancer |
| Pierce et al., 2010[250], Hess et al., 2012[251] | No members of vulnerable population(s) reported |  | Cardiovascular Health (Heart Disease, Stroke, etc…) |
| Pignone et al., 2000[252], Kim et al., 2005[253], Griffith et al., 2008a[254], Griffith et al., 2008b[255] | No (but they were included within a diverse population) | Older Age; Race and Ethnicity; Lower Education or Income | Colorectal Cancer |
| Pignone et al., 2004[256], Sheridan et al., 2006[257], Sheridan et al., 2009[258], Sheridan et al., 2010[259], Sheridan et al., 2011[260] | No (but they were included within a diverse population) | Race and Ethnicity; Lower Education or Income; Numeracy | Cardiovascular Health (Heart Disease, Stroke, etc…) |
| Raats et al., 2008[261] | No members of vulnerable population(s) reported |  | Prostate Cancer; Reproductive Health; Depression; Down Syndrome; Breast Cancer; Cardiovascular Health (Heart Disease, Stroke, etc…) |
| Raynes-Greenow et al., 2009[262], Raynes-Greenow et al., 2010[263] | No members of vulnerable population(s) reported |  | Reproductive Health |
| Rothert et al., 1997[264], Holmes-Rovner et al., 1999[265] | No members of vulnerable population(s) reported |  | Reproductive Health |
| Ruthman & Ferrans, 2004[266] | No (but they were included within a diverse population) | Older Age | Prostate Cancer |
| Saver et al., 2007[267] | No members of vulnerable population(s) reported |  | Reproductive Health |
| Sawka et al., 1998[268], Goel et al., 2001[269] | No (but they were included within a diverse population) | Older Age; Lower Education or Income | Breast Cancer |
| Schackmann et al., 2013[270] | No (but they were included within a diverse population) | Race and Ethnicity; Lower Education or Income | Breast Cancer; Ovarian Cancer |
| Schapira & VanRuiswyk, 2000[271] | No (but they were included within a diverse population) | Older Age; Lower Education or Income | Prostate Cancer |
| Schapira et al., 1997[272] | No (but they were included within a diverse population) | Race and Ethnicity; Lower Education or Income | Prostate Cancer |
| Schapira et al., 2007[273] | No members of vulnerable population(s) reported |  | Reproductive Health |
| Schonberg et al., 2014[274] | Yes | Older Age | Breast Cancer |
| Schroy et al., 2011[275], Schroy et al., 2012[276], Schroy et al., 2014[277] | No (but they were included within a diverse population) | Race and Ethnicity; Lower Literacy | Colorectal Cancer |
| Schwalm et al., 2012[278] | No members of vulnerable population(s) reported |  | Cardiovascular Health (Heart Disease, Stroke, etc…) |
| Schwartz, L.M. et al., 2009[279] | No members of vulnerable population(s) reported |  | Cardiovascular Health (Heart Disease, Stroke, etc…) |
| Schwartz, M.D. et al., 2009[280] | No members of vulnerable population(s) reported |  | Breast Cancer |
| Sepucha et al., 2009[281] | No (but they were included within a diverse population) | Older Age; Lower Education or Income | Breast Cancer |
| Shaffer et al., 2013[282] | No members of vulnerable population(s) reported |  | Breast Cancer |
| Sheppard et al., 2010[283] | Yes |  | Breast Cancer |
| Sheridan et al., 2004[284] | No (but they were included within a diverse population) | Older Age; Race and Ethnicity | Prostate Cancer |
| Sherman et al., 2014[285] | No members of vulnerable population(s) reported |  | Breast Cancer; Ductal Carcinoma In Situ (DCIS) |
| Shorten et al., 2004[286], Shorten et al., 2005[287] | Yes | Lower Education or Income | Reproductive Health |
| Simon et al., 2012[288] | Yes | Mental Health Condition(s) | Depression; Acute Low Back Pain |
| Smith et al., 2008[289], Trevena et al., 2008[290], Smith et al., 2009[291], Smith et al., 2010[292] | Yes | Lower Literacy | Colorectal Cancer |
| Solberg et al., 2010[293] | No members of vulnerable population(s) reported |  | Reproductive Health |
| Spunt et al., 1996[294], Deyo et al., 2000[295], Phelan et al., 2001[296] | No (but they were included within a diverse population) | Older Age | Low Back Problems (Herniated Disks; Spinal Stenosis; and other diagnoses) |
| Sridhar et al., 2015[297] | No members of vulnerable population(s) reported |  | Reproductive Health |
| Stacey et al., 2003[298] | No members of vulnerable population(s) reported |  | Breast Cancer |
| Stacey et al., 2014[299] | No members of vulnerable population(s) reported |  | Knee Osteoarthritis |
| Stalmeier & Roosmalen, 2009[300] | No members of vulnerable population(s) reported |  | Breast Cancer; Ovarian Cancer |
| Stalmeier & Roosmalen, 2009[300] | No members of vulnerable population(s) reported |  | Breast Cancer; Ovarian Cancer |
| Steckelberg et al., 2004[301], Steckelberg et al., 2011[302] | No members of vulnerable population(s) reported |  | Colorectal Cancer |
| Stein et al., 2013[303] | No members of vulnerable population(s) reported |  | Advance Care Planning; Cancer |
| Stiggelbout et al., 2008[304] | No members of vulnerable population(s) reported |  | Abdominal Aortic Aneurysm |
| Stirling et al., 2012[305] | Yes | Lower Education or Income; Mental Health Condition(s) | Dementia |
| Sudore et al., 2014[306] | Yes | Older Age; Race and Ethnicity; Lower Education or Income; Lower Literacy; Mental Health Condition(s) | Advance Care Planning |
| Tan et al., 2012[307], Tan et al., 2014[308] | No members of vulnerable population(s) reported |  | Psoriasis |
| Taylor et al., 2006[100], Dorfman et al., 2010[309], Kassan et al., 2012[310], Taylor et al., 2013[311], Williams et al., 2013[312] | No (but they were included within a diverse population) | Race and Ethnicity; Lower Education or Income; Other: Internet Access | Prostate Cancer |
| Taylor et al., 2006[100], Dorfman et al., 2010[309], Kassan et al., 2012[310], Taylor et al., 2013[311], Williams et al., 2013[312] | No (but they were included within a diverse population) | Race and Ethnicity; Lower Education or Income; Other: Internet Access | Prostate Cancer |
| Taylor et al., 2006[100], Dorfman et al., 2010[309], Kassan et al., 2012[310], Taylor et al., 2013[311], Williams et al., 2013[312] | No (but they were included within a diverse population) | Race and Ethnicity; Lower Education or Income; Other: Internet Access | Prostate Cancer |
| Thomson et al., 2002[313], Kaner et al., 2007[314], Thomson et al., 2007[315] | No (but they were included within a diverse population) | Older Age | Cardiovascular Health (Heart Disease, Stroke, etc…) |
| Tiller et al., 2003[316], Tiller et al., 2006[317] | No members of vulnerable population(s) reported |  | Ovarian Cancer |
| van der Krieke et al., 2012[318] | Yes | Mental Health Condition(s) | Schizophrenia |
| van Peperstraten et al., 2010a[319], van Peperstraten et al., 2010b[320] | No members of vulnerable population(s) reported |  | Reproductive Health |
| van Tol-Geerdink et al., 2006[321], van Tol-Geerdink et al., 2008[322] | No (but they were included within a diverse population) | Older Age | Prostate Cancer |
| van Tol-Geerdink et al., 2013[323] | No (but they were included within a diverse population) | Older Age | Prostate Cancer |
| Vandemheen et al., 2009[324], Vandemheen et al., 2010[325] | No members of vulnerable population(s) reported |  | Cystic Fibrosis |
| Volandes et al., 2009a[326], Volandes et al., 2009b[327], Volandes et al., 2010[328] | No (but they were included within a diverse population) | Older Age; Race and Ethnicity; Lower Education or Income | Advance Care Planning; Dementia |
| Volk et al., 1999[329], Volk et al., 2003[330] | No members of vulnerable population(s) reported |  | Prostate Cancer |
| Volk et al., 2008[331] | No (but they were included within a diverse population) | Race and Ethnicity; Lower Literacy | Prostate Cancer |
| Volk et al., 2014[332] | No (but they were included within a diverse population) | Race and Ethnicity; Lower Education or Income | Lung Cancer |
| Wakefield et al., 2008a[333] | No members of vulnerable population(s) reported |  | Colorectal Cancer |
| Wakefield et al., 2011[334], Watts et al., 2013[335] | No (but they were included within a diverse population) | Older Age | Prostate Cancer |
| Weng et al., 2007[336] | Yes | Race and Ethnicity; Lower Education or Income | Knee Osteoarthritis |
| Willemsen et al., 2006[337] | No (but they were included within a diverse population) | Lower Education or Income | Smoking |
| Wilson et al., 2015[338] | No members of vulnerable population(s) reported |  | Intensive Care Unit; Cardiovascular Health (Heart Disease, Stroke, etc…) |
| Wong et al., 2006[339] | No (but they were included within a diverse population) |  | Reproductive Health |
| Wong et al., 2012[340] | Yes | Older Age | Breast Cancer |
| Wright et al., 2002[341], Wright et al., 2004[342] | No members of vulnerable population(s) reported |  | Cardiovascular Health (Heart Disease, Stroke, etc…) |
| Zapka et al., 2004[343] | No members of vulnerable population(s) reported |  | Colorectal Cancer |

**References for Articles Describing Included Projects**

28. Allen JD, Mohllajee AP, Shelton RC, Drake BF, Mars DR: **A computer-tailored intervention to promote informed decision making for prostate cancer screening among African American men.** *Am J Mens Health* 2009, **3**:340–51.

29. Anderson C, Carter J, Nattress K, Beale P, Philp S, Harrison J, Juraskova I: **“The booklet helped me not to panic”: A pilot of a decision aid for asymptomatic women with ovarian cancer and with rising CA-125 levels**. *Int J Gynecol Cancer* 2011, **21**:737–743.

30. Arterburn DE, Westbrook EO, Bogart TA, Sepucha KR, Bock SN, Weppner WG: **Randomized trial of a video-based patient decision aid for bariatric surgery.** *Obesity* 2011, **19**:1669–75.

31. Au AHY, Lam WWT, Chan MCM, Or AYM, Kwong A, Suen D, Wong AL, Juraskova I, Wong TWT, Fielding R: **Development and pilot-testing of a Decision Aid for use among Chinese women facing breast cancer surgery**. *Heal Expect* 2011, **14**:405–416.

32. Lam WWT, Chan M, Or A, Kwong A, Suen D, Fielding R: **Reducing treatment decision conflict difficulties in breast cancer surgery: a randomized controlled trial.** *J Clin Oncol* 2013, **31**:2879–2885.

33. Auvinen A, Hakama M, Ala-Opas M, Vornanen T, Leppilahti M, Salminen P, Tammela TL: **A randomized trial of choice of treatment in prostate cancer: the effect of intervention on the treatment chosen**. *BJU Int* 2004, **93**:52–6.

34. Bailey DB, Bann C, Bishop E, Guarda S, Barnum L, Roche M: **Can a decision aid enable informed decisions in neonatal nursery recruitment for a fragile X newborn screening study?** *Genet Med* 2012, **15**:299–306.

35. Bailey DB, Lewis MA, Harris SL, Grant T, Bann C, Bishop E, Roche M, Guarda S, Barnum L, Powell C, Therrell BL: **Design and evaluation of a decision aid for inviting parents to participate in a fragile X newborn screening pilot study**. *J Genet Couns* 2013, **22**:108–117.

36. Barnieh L, McLaughlin K, Manns BJ, Klarenbach S, Yilmaz S, Taub K, Hemmelgarn BR: **Evaluation of an education intervention to increase the pursuit of living kidney donation: a randomized controlled trial.** *Prog Transplant* 2011, **21**:36–42.

37. Barry MJ, Fowler FJ, Mulley a G, Henderson J V, Wennberg JE: **Patient reactions to a program designed to facilitate patient participation in treatment decisions for benign prostatic hyperplasia.** *Med Care* 1995, **33**:771–782.

38. Barry MJ, Cherkin DC, YuChiao C, Fowler FJ, Skates S: **A randomized trial of a multimedia shared decision-making program for men facing a treatment decision for benign prostatic hyperplasia**. *Dis Manag Clin Outcomes* 1997, **1**:5–14.

39. Piercy GB, Deber R, Trachtenberg J, Ramsey EW, Norman RW, Goldenberg SL, Nickel JC, Elhilali M, Perrault JP, Kraetschmer N, Sharpe N: **Impact of a shared decision-making program on patients with benign prostatic hyperplasia**. *Urology* 1999, **53**:913–920.

40. Rovner DR, Wills CE, Bonham V, Williams G, Lillie J, Kelly-Blake K, Williams M V, Holmes-Rovner M: **Decision aids for benign prostatic hyperplasia: applicability across race and education**. *Med Decis Mak* 2004, **24**:359–366.

41. Holmes-Rovner M, Price C, Rovner DR, Kelly-Blake K, Lillie J, Wills C, Bonham VL: **Men’s theories about benign prostatic hyperplasia and prostate cancer following a benign prostatic hyperplasia decision aid**. *J Gen Intern Med* 2006, **21**:56–60.

42. Wills CE, Holmes-Rovner M, Rovner D, Lillie J, Kelly-Blake K, Bonham V, Williams G: **Treatment preference patterns during a videotape decision aid for benign prostatic hyperplasia (BPH)**. *Patient Educ Couns* 2006, **61**:16–22.

43. Pylar J, Wills CE, Lillie J, Rovner DR, Kelly-Blake K, Holmes-Rovner M: **Men’s interpretations of graphical information in a videotape decision aid**. *Heal Expect* 2007, **10**:184–193.

44. Bass SB, Gordon TF, Ruzek SB, Wolak C, Ruggieri D, Mora G, Rovito MJ, Britto J, Parameswaran L, Abedin Z, Ward S, Paranjape A, Lin K, Meyer B, Pitts K: **Developing a computer touch-screen interactive colorectal screening decision aid for a low-literacy African American population: lessons learned.** *Health Promot Pract* 2013, **14**:589–98.

45. Bastian LA, McBride CM, Fish L, Lyna P, Farrell D, Lipkus IM, Rimer BK, Siegler IC: **Evaluating participants’ use of a hormone replacement therapy decision-making intervention**. *Patient Educ Couns* 2002, **48**:283–291.

46. McBride CM, Bastian LA, Halabi S, Fish L, Lipkus IM, Bosworth HB, Rimer BK, Siegler IC: **A tailored intervention to aid decisionmaking about hormone replacement therapy**. *Am J Public Health* 2002, **92**:1112–1114.

47. Becker H, Stuifbergen AK, Dormire SL: **The effects of hormone therapy decision support for women with mobility impairments**. *Health Care Women Int* 2009, **30**:845–854.

48. Bekker H, Thornton J, Lilleyman J, MacIntosh M, Maule A, Airey C, Michie S, Pearman A, Robinson M, Connelly J, Hewison J: **Informed decision making: an annotated bibliography and systematic review**. *Health Technol Assess (Rockv)* 1999, **3**:168.

49. Bekker HL, Hewison J, Thornton JG: **Applying decision analysis to facilitate informed decision making about prenatal diagnosis for Down syndrome: A randomised controlled trial**. *Prenat Diagn* 2004, **24**:265–275.

50. Berman L, Curry L, Goldberg C, Gusberg R, Fraenkel L: **Pilot testing of a decision support tool for patients with abdominal aortic aneurysms**. *J Vasc Surg* 2011, **53**:285–292.

51. Berry DL, Halpenny B, Wolpin S, Davison BJ, Ellis WJ, Lober WB, McReynolds J, Wulff J: **Development and evaluation of the personal Patient Profile-Prostate (P3P), a Web-based decision support system for men newly diagnosed with localized prostate cancer**. *J Med Internet Res* 2010, **12**:e67.

52. Berry DL, Halpenny B, Hong F, Wolpin S, Lober WB, Russell KJ, Ellis WJ, Govindarajulu U, Bosco J, Davison BJ, Bennett G, Terris MK, Barsevick A, Lin DW, Yang CC, Swanson G: **The Personal Patient Profile-Prostate decision support for men with localized prostate cancer: a multi-center randomized trial**. *Urol Oncol* 2013, **31**:1012–21.

53. Breslin M, Mullan RJ, Montori VM: **The design of a decision aid about diabetes medications for use during the consultation with patients with type 2 diabetes**. *Patient Educ Couns* 2008, **73**:465–472.

54. Mullan RJ, Montori VM, Shah ND, Christianson TJH, Bryant SC, Guyatt GH, Perestelo-Perez LI, Stroebel RJ, Yawn BP, Yapuncich V, Breslin MA, Pencille L, Smith SA: **The diabetes mellitus medication choice decision aid: a randomized trial.** *Arch Intern Med* 2009, **169**:1560–8.

55. Brink SG, Birney AJ, McFarren AE: **Charting your course: formative evaluation of a prostate cancer treatment decision aid**. *Int Electron J Health Educ* 2000, **3**:44–54.

56. Fagerlin A, Rovner D, Stableford S, Jentoft C, Wei JT, Holmes-Rovner M: **Patient Education Materials about the Treatment of Early-Stage Prostate Cancer: A Critical Review**. *Annals of Internal Medicine* 2004:721–728.

57. Brohan E, Evans-Lacko S, Henderson C, Murray J, Slade M, Thornicroft G: **Disclosure of a mental health problem in the employment context: qualitative study of beliefs and experiences.** *Epidemiol Psychiatr Sci* 2014, **23**:289–300.

58. Brohan E, Henderson C, Slade M, Thornicroft G: **Development and preliminary evaluation of a decision aid for disclosure of mental illness to employers**. *Patient Educ Couns* 2014, **94**:238–242.

59. Chewning B, Mosena P, Wilson D, Erdman H, Potthoff S, Murphy A, Kennedy Kuhnen K: **Evaluation of a computerized contraceptive decision aid for adolescent patients**. *Patient Educ Couns* 1999, **38**:227–239.

60. Chiew KS, Shepherd H, Vardy J, Tattersall MHN, Butow PN, Leighl NB: **Development and evaluation of a decision aid for patients considering first-line chemotherapy for metastatic breast cancer**. *Heal Expect* 2008, **11**:35–45.

61. Consoli SM, Said M Ben, Jean J, Menard J, Plouin PF, Chatellier G: **Benefits of a computer-assisted education program for hypertensive patients compared with standard education tools**. *Patient Educ Couns* 1995, **26**:343–347.

62. Costanza ME, Luckmann RS, Rosal M, White MJ, LaPelle N, Partin M, Cranos C, Leung KG, Foley C: **Helping men make an informed decision about prostate cancer screening: A pilot study of telephone counseling**. *Patient Educ Couns* 2011, **82**:193–200.

63. Cox CE, Lewis CL, Hanson LC, Hough CL, Kahn JM, White DB, Song M-K, Tulsky J a., Carson SS: **Development and pilot testing of a decision aid for surrogates of patients with prolonged mechanical ventilation**. *Crit Care Med* 2012, **40**:2327–2334.

64. Dales RE, O’Connor A, Hebert P, Sullivan K, McKim D, Llewellyn-Thomas H: **Intubation and mechanical ventilation for COPD: Development of an instrument to elicit patient preferences**. *Chest* 1999, **116**:792–800.

65. Wilson KG, Aaron SD, Vandemheen KL, Hebert PC, McKim DA, Fiset V, Graham ID, Sevigny E, O’Connor AM: **Evaluation of a decision aid for making choices about intubation and mechanical ventilation in chronic obstructive pulmonary disease**. *Patient Educ Couns* 2005, **57**:88–95.

66. Davis TC, Fredrickson DD, Arnold C, Murphy PW, Herbst M, Bocchini JA: **A polio immunization pamphlet with increased appeal and simplified language does not improve comprehension to an acceptable level**. *Patient Educ Couns* 1998, **33**:25–37.

67. Dillard AJ, Fagerlin A, Cin SD, Zikmund-Fisher BJ, Ubel PA: **Narratives that address affective forecasting errors reduce perceived barriers to colorectal cancer screening**. *Soc Sci Med* 2010, **71**:45–52.

68. Dodin S, Daudelin G, Tetroe J, O’Connor A, Legare F: **Prise de decision en matière d’hormonothérapie de remplacement [Making a decision about hormone replacement therapy. A randomized controlled trial]**. *Can Fam Physician* 2001, **47**:1586–1593.

69. Dolan JG, Frisina S: **Randomized Controlled Trial of a Patient Decision Aid for Colorectal Cancer Screening**. *Med Decis Mak* 2002, **22**:125–139.

70. Dolan JG, Veazie PJ, Russ AJ: **Development and initial evaluation of a treatment decision dashboard**. *BMC Med Inform Decis Mak* 2013, **13**:51.

71. Dowding D, Swanson V, Bland R, Thomson P, Mair C, Morrison A, Taylor A, Beechey C, Simpson R, Niven K: **The development and preliminary evaluation of a decision aid based on decision analysis for two treatment conditions: Benign Prostatic Hyperplasia and Hypertension**. *Patient Educ Couns* 2004, **52**:209–215.

72. Thomson P, Dowding D, Swanson V, Bland R, Mair C, Morrison A, Taylor A, Beechey C, Niven C a: **A computerised guidance tree (decision aid) for hypertension, based on decision analysis: development and preliminary evaluation.** *Eur J Cardiovasc Nurs* 2006, **5**:146–9.

73. Drake BF, Shelton RC, Gilligan T, Allen JD: **A church-based intervention to promote informed decision making for prostate cancer screening among African American men.** *J Natl Med Assoc* 2010, **102**:164–71.

74. Durand M-A: **Amniocentesis dilemma: needs assessment, development and field-testing of a theory-based decision support intervention**. Cardiff University; 2009.

75. Durand M-A, Stiel M, Boivin J, Elwyn G: **Information and decision support needs of parents considering amniocentesis: Interviews with pregnant women and health professionals**. *Heal Expect* 2010, **13**:125–38.

76. Durand MA, Wegwarth O, Boivin J, Elwyn G: **Design and usability of heuristic-based deliberation tools for women facing amniocentesis**. *Heal Expect* 2012, **15**:32–48.

77. Elit LM, Levine MN, Gafni a, Whelan TJ, Doig G, Streiner DL, Rosen B: **Patients’ preferences for therapy in advanced epithelial ovarian cancer: development, testing, and application of a bedside decision instrument.** *Gynecol Oncol* 1996, **62**:329–35.

78. Emmett CL, Murphy DJ, Patel RR, Fahey T, Jones C, Ricketts IW, Gregor P, Macleod M, Montgomery AA: **Decision-making about mode of delivery after previous caesarean section: Development and piloting of two computer-based decision aids**. *Heal Expect* 2007, **10**:161–172.

79. Montgomery AA, Emmett CL, Fahey T, Jones C, Ricketts I, Patel RR, Peters TJ, Murphy DJ: **Two decision aids for mode of delivery among women with previous caesarean section: randomised controlled trial**. *BMJ* 2007:1305.

80. Frost J, Shaw A, Ontgomery A, Murphy D: **Women’s views on the use of decision aids for decision making about the method of delivery following a previous caesarean section: Qualitative interview study**. *BJOG* 2009, **116**:896–905.

81. Hollinghurst S, Emmett C, Peters TJ, Watson H, Fahey T, Murphy DJ, Montgomery A: **Economic evaluation of the DiAMOND randomized trial: cost and outcomes of 2 decision aids for mode of delivery among women with a previous cesarean section**. *Med Decis Making* 2010, **30**:453–463.

82. Evans R, Elwyn G, Edwards A, Watson E, Austoker J, Grol R: **Toward a model for field-testing patient decision-support technologies: A qualitative field-testing study**. *J Med Internet Res* 2007, **9**:e21.

83. Evans R, Joseph-Williams N, Edwards A, Newcombe RG, Wright P, Kinnersley P, Griffiths J, Jones M, Williams J, Grol R, Elwyn G: **Supporting informed decision making for prostate specific antigen (PSA) testing on the web: An online randomized controlled trial**. *J Med Internet Res* 2010, **12**:e27.

84. Joseph-Williams N, Evans R, Edwards A, Newcombe RG, Wright P, Grol R, Elwyn G: **Supporting informed decision making online in 20 minutes: an observational web-log study of a PSA test decision aid**. *J Med Internet Res* 2010, **12**:e15.

85. Fagerlin A, Zikmund-Fisher BJ, Smith DM, Nair V, Derry HA, McClure JB, Greene S, Stark A, Alford SH, Lantz P, Hayes DF, Wiese C, Zweig SC, Pitsch R, Jankovic A, Ubel PA: **Women’s decisions regarding tamoxifen for breast cancer prevention: Responses to a tailored decision aid**. *Breast Cancer Res Treat* 2010, **119**:613–620.

86. Fagerlin A, Dillard AJ, Smith DM, Zikmund-Fisher BJ, Pitsch R, McClure JB, Greene S, Alford SH, Nair V, Hayes DF, Wiese C, Ubel PA: **Women’s interest in taking tamoxifen and raloxifene for breast cancer prevention: Response to a tailored decision aid**. *Breast Cancer Res Treat* 2011, **127**:681–688.

87. Banegas MP, McClure JB, Barlow WE, Ubel PA, Smith DM, Zikmund-Fisher BJ, Greene SM, Fagerlin A: **Results from a randomized trial of a web-based, tailored decision aid for women at high risk for breast cancer**. *Patient Educ Couns* 2013, **91**:364–371.

88. Korfage IJ, Fuhrel-Forbis A, Ubel PA, Zikmund-Fisher BJ, Greene SM, McClure JB, Smith DM, Alford SH, Fagerlin A: **Informed choice about breast cancer prevention: randomized controlled trial of an online decision aid intervention**. *Breast Cancer Res* 2013, **15**:R74.

89. Farnworth A, Robson SC, Thomson RG, Watson DB, Murtagh MJ: **Decision support for women choosing mode of delivery after a previous caesarean section: A developmental study**. *Patient Educ Couns* 2008, **71**:116–124.

90. Feldman-Stewart D, Brundage MD, Hayter C, Groome P, Nickel JC, Downes H, Mackillop WJ: **What questions do patients with curable prostate cancer want answered?** *Med Decis Mak* 2000, **20**:7–19.

91. Feldman-stewart D, Brundage MD, Nickel JC, Mackillop WJ: **The information required by patients with early-stage prostate cancer in choosing their treatment**. *BJU Int* 2001, **87**:218–223.

92. Feldman-Stewart D, Brundage MD, Van Manen L: **A decision aid for men with early stage prostate cancer: Theoretical basis and a test by surrogate patients**. *Heal Expect* 2001, **4**:221–234.

93. Feldman-Stewart D, Brundage MD, Van Manen L, Skarsgard D, Siemens R: **Evaluation of a question-and-answer booklet on early-stage prostate-cancer**. *Patient Educ Couns* 2003, **49**:115–124.

94. Feldman-Stewart D, Brundage MD, Van Manen L, Svenson O: **Patient-focussed decision-making in early-stage prostate cancer: Insights from a cognitively based decision aid**. *Heal Expect* 2004, **7**:126–141.

95. Feldman-Stewart D, Brennenstuhl S, Brundage MD, Roques T: **An explicit values clarification task: Development and validation**. *Patient Educ Couns* 2006, **63**:350–356.

96. Feldman-Stewart D, Brundage M, Siemens R, Skarsgard D: **A randomized controlled trial comparing two educational booklets on prostate cancer**. *Can J Urol* 2006, **13**:3321–3326.

97. Feldman-Stewart D, Tong C, Siemens R, Alibhai S, Pickles T, Robinson J, Brundage MD: **The Impact of Explicit Values Clarification Exercises in a Patient Decision Aid Emerges After the Decision Is Actually Made: Evidence From a Randomized Controlled Trial**. *Medical Decision Making* 2012:616–626.

98. Fetters MD, Ivankova N V, Ruffin MT, Creswell JW, Power D: **Developing a Web site in primary care.** *Fam Med* 2004, **36**:651–9.

99. Ruffin MT, Fetters MD, Jimbo M: **Preference-based electronic decision aid to promote colorectal cancer screening: results of a randomized controlled trial.** *Prev Med (Baltim)* 2007, **45**:267–73.

100. Taylor KL, Davis JL, Turner RO, Johnson L, Schwartz MD, Kerner JF, Leak C: **Educating African American men about the prostate cancer screening dilemma: a randomized intervention.** *Cancer Epidemiol Biomarkers Prev* 2006, **15**:2179–88.

101. Taylor KL, Davis KM, Lamond T, Williams RM, Schwartz MD, Lawrence W, Feng S, Brink S, Birney A, Lynch J, Regan J, Dritschilo A: **Use and evaluation of a CD-ROM-based decision aid for prostate cancer treatment decisions.** *Behav Med* 2010, **36**:130–40.

102. Fiset V, O’Connor AM, Evans W, Graham I, Degrasse C, Logan J: **Development and evaluation of a decision aid for patients with stage IV non-small cell lung cancer**. *Heal Expect* 2000, **3**:125–136.

103. Fleisher L, Buzaglo J, Collins M, Millard J, Miller SM, Egleston BL, Solarino N, Trinastic J, Cegala DJ, Benson AB, Schulman KA, Weinfurt KP, Sulmasy D, Diefenbach MA, Meropol NJ: **Using health communication best practices to develop a web-based provider -patient communication aid: The CONNECT[TM] study**. *Patient Educ Couns* 2008, **71**:378–387.

104. Meropol NJ, Egleston BL, Buzaglo JS, Balshem A, Benson AB, Cegala DJ, Cohen RB, Collins M, Diefenbach MA, Miller SM, Fleisher L, Millard JL, Ross EA, Schulman KA, Silver A, Slater E, Solarino N, Sulmasy DP, Trinastic J, Weinfurt KP: **A Web-based communication aid for patients with cancer: The CONNECT Study**. *Cancer* 2013, **119**:1437–1445.

105. Flood AB, Wennberg JE, Nease Jr. RF, Fowler Jr. FJ, Ding J, Hynes LM: **The importance of patient preference in the decision to screen for prostate cancer. Prostate Patient Outcomes Research Team**. *J Gen Intern Med* 1996, **11**:342–349.

106. Partin MR, Nelson D, Radosevich D, Nugent S, Flood AB, Dillon N, Holtzman J, Haas M, Wilt TJ: **Randomized trial examining the effect of two prostate cancer screening educational interventions on patient knowledge, preferences, and behaviors**. *J Gen Intern Med* 2004, **19**:835–842.

107. Partin MR, Nelson D, Flood AB, Friedemann-Sánchez G, Wilt TJ: **Who uses decision aids? Subgroup analyses from a randomized controlled effectiveness trial of two prostate cancer screening decision support interventions.** *Heal Expect* 2006, **9**:285–95.

108. Fortin JM, Hirota LK, Bond BE, O’Connor AM, Col NF: **Identifying patient preferences for communicating risk estimates: a descriptive pilot study**. *BMC Med Inform Decis Mak* 2001, **1**:2.

109. Bond B, Hirota L, Fortin J, Col N: **Women like me: Reflections on health and hormones from women treated for breast cancer**. *J Psychosoc Oncol* 2002, **20**:39–56.

110. Col NF, Ngo L, Fortin JM, Goldberg RJ, O’Connor AM: **Can computerized decision support help patients make complex treatment decisions? A randomized controlled trial of an individualized menopause decision aid.** *Med Decis Making* 2007, **27**:585–598.

111. Fraenkel L, Street RL, Fried TR: **Development of a tool to improve the quality of decision making in atrial fibrillation**. *BMC Med Inform Decis Mak* 2011, **11**:59.

112. Fraenkel L, Street Jr. RL, Towle V, O’Leary JR, Iannone L, Van Ness PH, Fried TR: **A pilot randomized controlled trial of a decision support tool to improve the quality of communication and decision‐making in individuals with atrial fibrillation.** *J Am Geriatr Soc* 2012, **60**:1434–1441.

113. Fraenkel L, Peters E, Charpentier P, Olsen B, Errante L, Schoen RT, Reyna V: **Decision tool to improve the quality of care in rheumatoid arthritis.** *Arthritis Care Res* 2012, **64**:977–85.

114. Fraenkel L, Matzko CK, Webb DE, Oppermann B, Charpentier P, Peters E, Reyna V, Newman ED: **Use of decision support for improved knowledge, values clarification, and informed choice in patients with rheumatoid arthritis**. *Arthritis Care Res* 2015, **67**:1496–1502.

115. Frank C, Pichora D, Suurdt J, Heyland D: **Development and use of a decision aid for communication with hospitalized patients about cardiopulmonary resuscitation preference**. *Patient Educ Couns* 2010, **79**:130–133.

116. French RS, Cowan FM, Wellings K, Dowie J: **The development of a multi-criteria decision analysis aid to help with contraceptive choices: My Contraception Tool.** *J Fam Plann Reprod Health Care* 2014, **40**:96–101.

117. Frosch DL, Kaplan RM, Felitti V: **Evaluation of two methods to facilitate shared decision making for men considering the prostate-specific antigen test**. *J Gen Intern Med* 2001, **16**:391–398.

118. Frosch DL, Kaplan RM, Felitti VJ: **A Randomized Controlled Trial Comparing Internet and Video to Facilitate Patient Education for Men Considering the Prostate Specific Antigen Test**. *J Gen Intern Med* 2003, **18**:781–787.

119. Frosch DL, Bhatnagar V, Tally S, Hamori CJ, Kaplan RM: **Internet patient decision support: a randomized controlled trial comparing alternative approaches for men considering prostate cancer screening.** *Arch Intern Med* 2008, **168**:363–9.

120. Bhatnagar V, Frosch DL, Tally SR, Hamori CJ, Lenert L, Kaplan RM: **Evaluation of an Internet-based disease trajectory decision tool for prostate cancer screening**. *Value Heal* 2009, **12**:101–108.

121. Frosch DL, Légaré F, Mangione CM: **Using decision aids in community-based primary care: A theory-driven evaluation with ethnically diverse patients**. *Patient Educ Couns* 2008, **73**:490–496.

122. Gallo AM, Wilkie D, Suarez M, Labotka R, Molokie R, Thompson A, Hershberger P, Johnson B: **Reproductive decisions in people with sickle cell disease or sickle cell trait.** *West J Nurs Res* 2010, **32**:1073–90.

123. Gallo AM, Wilkie DJ, Wang E, Labotka RJ, Molokie RE, Stahl C, Hershberger PE, Zhao Z, Suarez ML, Johnson B, Pullum C, Angulo R, Thompson A: **Evaluation of the SCKnowIQ Tool and Reproductive CHOICES Intervention Among Young Adults With Sickle Cell Disease or Sickle Cell Trait.** *Clin Nurs Res* 2013, **23**:421–441.

124. Wilkie DJ, Gallo AM, Yao Y, Molokie RE, Stahl C, Hershberger PE, Zhao Z, Suarez ML, Labotka RJ, Johnson B, Angulo R, Angulo V, Carrasco J, Shuey D, Pelligra S, Wang E, Rogers DT, Thompson A a: **Reproductive health choices for young adults with sickle cell disease or trait: randomized controlled trial immediate posttest effects.** *Nurs Res* 2013, **62**:352–61.

125. Garvelink MM, Ter Kuile MM, Fischer MJ, Louwé LA, Hilders CGJM, Kroep JR, Stiggelbout AM: **Development of a Decision Aid about fertility preservation for women with breast cancer in the Netherlands**. *J Psychosom Obstet Gynecol* 2013, **34**:170–178.

126. Green MJ, Fost N: **An interactive computer program for educating and counseling patients about genetic susceptibility to breast cancer**. *J Cancer Educ* 1997, **12**:204–208.

127. Lerman C, Biesecker B, Benkendorf JL, Kerner J, Gomez-Caminero a, Hughes C, Reed MM: **Controlled trial of pretest education approaches to enhance informed decision-making for BRCA1 gene testing.** *J Natl Cancer Inst* 1997, **89**:148–57.

128. Green MJ, Biesecker BB, McInerney a M, Mauger D, Fost N: **An interactive computer program can effectively educate patients about genetic testing for breast cancer susceptibility.** *Am J Med Genet* 2001, **103**:16–23.

129. Schwartz MD, Benkendorf J, Lerman C, Isaacs C, Ryan-Robertson A, Johnson L: **Impact of educational print materials on knowledge, attitudes, and interest in BRCA1/BRCA2: Testing among Ashkenazi Jewish women**. *Cancer* 2001, **92**:932–940.

130. Green MJ, Peterson SK, Baker MW, Harper GR, Friedman LC, Rubinstein WS, Manger DT: **Effect of a computer-based decision aid on knowledge, perceptions, and intentions about genetic testing for breast cancer susceptibility - A randomized controlled trial**. *JAMA* 2004, **292**:442–452.

131. Green MJ, Peterson SK, Baker MW, Friedman LC, Harper GR, Rubinstein WS, Peters J a, Mauger DT: **Use of an educational computer program before genetic counseling for breast cancer susceptibility: effects on duration and content of counseling sessions.** *Genet Med* 2005, **7**:221–229.

132. Green MJ, Levi BH: **Development of an interactive computer program for advance care planning**. *Heal Expect* 2009, **12**:60–69.

133. Hossler C, Levi BH, Simmons Z, Green MJ: **Advance care planning for patients with ALS: feasibility of an interactive computer program.** *Amyotroph Lateral Scler* 2011, **12**:172–177.

134. Markham SA, Levi BH, Green MJ, Schubart JR: **Use of a Computer Program for Advance Care Planning with African American Participants**. *J Natl Med Assoc* 2015, **107**:26–32.

135. Gustafson D, Wise M, McTavish F, Taylor JO, Wolberg W, Stewart J, Smalley R, Bosworth K: **Development and Pilot Evaluation of a Computer-Based Support System for Women with Breast Cancer**. *J Psychosoc Oncol* 1994, **11**:69–93.

136. Gustafson DH, Hawkins R, Pingree S, McTavish F, Arora NK, Mendenhall J, Cella DF, Serlin RC, Apantaku FM, Stewart J, Salner A: **Effect of computer support on younger women with breast cancer.** *J Gen Intern Med* 2001, **16**:435–45.

137. Wise M, Han JY, Shaw B, McTavish F, Gustafson DH: **Effects of using online narrative and didactic information on healthcare participation for breast cancer patients**. *Patient Educ Couns* 2008, **70**:348–356.

138. Hamann J, Langer B, Winkler V, Busch R, Cohen R, Leucht S, Kissling W: **Shared decision making for in-patients with schizophrenia**. *Acta Psychiatr Scand* 2006, **114**:265–273.

139. Hamann J, Cohen R, Leucht S, Busch R, Kissling W: **Shared decision-making and long-term outcome in schizophrenia treatment**. *J Clin Psychiatry* 2007, **68**:993–998.

140. Hawkins Virani AK, Creighton SM, Hayden MR: **Developing a comprehensive, effective patient-friendly website to enhance decision making in predictive testing for Huntington disease.** *Genet Med* 2013, **15**:466–72.

141. Heinrich E, de Nooijer J, Schaper NC, Schoonus-Spit MHG, Janssen MAJ, de Vries NK: **Evaluation of the web-based Diabetes Interactive Education Programme (DIEP) for patients with type 2 diabetes**. *Patient Educ Couns* 2012, **86**:172–178.

142. Henderson VA, Barr KL, An LC, Guajardo C, Newhouse W, Mase R, Heisler M: **Community-based participatory research and user-centered design in a diabetes medication information and decision tool.** *Prog Community Health Partnersh* 2013, **7**:171–84.

143. Hightow-Weidman LB, Fowler B, Kibe J, McCoy R, Pike E, Calabria M, Adimora A: **Healthmpowerment.org: Development of a theory-based HIV/STI website for young black MSM**. *AIDS Educ Prev* 2011, **23**:1–12.

144. Muessig KE, Pike EC, Fowler B, LeGrand S, Parsons JT, Bull SS, Wilson PA, Wohl DA, Hightow-Weidman LB: **Putting prevention in their pockets: developing mobile phone-based HIV interventions for black men who have sex with men.** *AIDS Patient Care STDS* 2013, **27**:211–22.

145. Muessig KE, Baltierra NB, Pike EC, LeGrand S, Hightow-Weidman LB: **Achieving HIV risk reduction through HealthMpowerment.org, a user-driven eHealth intervention for young Black men who have sex with men and transgender women who have sex with men.** *Digit Cult Educ* 2014, **6**:164–182.

146. Hightow-Weidman MD, MPH LB, Muessig PhD KE, Pike BS EC, LeGrand PhD S, Baltierra BS N, Rucker MPH AJ, Wilson PhD P: **HealthMpowerment.org: Building Community Through a Mobile-Optimized, Online Health Promotion Intervention**. *Heal Educ Behav* 2015, **42**:493.

147. Hill-Briggs F, Renosky R, Lazo M, Bone L, Hill M, Levine D, Brancati FL, Peyrot M: **Development and pilot evaluation of literacy-adapted diabetes and CVD education in urban, diabetic African Americans**. *J Gen Intern Med* 2008, **23**:1491–1494.

148. Schumann KP, Sutherland JA, Majid HM, Hill-Briggs F: **Evidence-Based Behavioral Treatments for Diabetes: Problem-Solving Therapy**. *Diabetes Spectr* 2011, **24**:64–69.

149. Majid H, Schumann K, Doswell A, Sutherland J, Hill Golden S, Stewart K, Hill-Briggs F: **Development and evaluation of the DECIDE to move! Physical activity educational video.** *Diabetes educ* 2012, **38**:855–9.

150. Hoffner B, Bauer-Wu S, Hitchcock-Bryan S, Powell M, Wolanski A, Joffe S: **“Entering a clinical trial: Is it right for you?” - A randomized study of the clinical trials video and its impact on the informed consent process**. *Cancer* 2012, **118**:1877–1883.

151. Holbrook A, Labiris R, Goldsmith CH, Ota K, Harb S, Sebaldt RJ: **Influence of decision aids on patient preferences for anticoagulant therapy: a randomized trial.** *CMAJ* 2007, **176**:1583–7.

152. Hollen P, PhD RN, Tyc V, Donnangelo S, Shannon S, RN MS, CPNP C, O’Laughlen M, Hinton I, Smolkin M, Petroni G: **A Substance Use Decision Aid for Medically at-Risk Adolescents: Results of a Randomized Controlled Trial for Cancer-Surviving Adolescents.** *Cancer Nurs* 2013:355–367.

153. Hollen PJ, Gralla RJ, Jones RA, Thomas CY, Brenin DR, Weiss GR, Schroen AT, Petroni GR: **A theory-based decision aid for patients with cancer: Results of feasibility and acceptability testing of DecisionKEYS for cancer**. *Support Care Cancer* 2013, **21**:889–899.

154. Holmes-Rovner M, Stableford S, Fagerlin A, Wei JT, Dunn RL, Ohene-Frempong J, Kelly-Blake K, Rovner DR: **Evidence-based patient choice: a prostate cancer decision aid in plain language**. *BMC Med Inform Decis Mak* 2005, **5**:16.

155. Hong C, Kim S, Curnew G, Schulman S, Pullenayegum E, Holbrook A: **Validation of a patient decision aid for choosing between dabigatran and warfarin for atrial fibrillation**. *J Popul Ther Clin Pharmacol* 2013, **20**:e229–e237.

156. Hooker GW, Leventhal K-G, DeMarco T, Peshkin BN, Finch C, Wahl E, Joines JR, Brown K, Valdimarsdottir H, Schwartz MD: **Longitudinal changes in patient distress following interactive decision aid use among BRCA1/2 carriers: a randomized trial.** *Med Decis Making* 2011, **31**:412–421.

157. Hope N, Rombauts L: **Can an educational DVD improve the acceptability of elective single embryo transfer? A randomized controlled study**. *Fertil Steril* 2010, **94**:489–495.

158. Hutchison C, Campbell S: **Evaluation of an information booklet for patients considering participation in phase I clinical trials in cancer**. *Eur J Cancer Care (Engl)* 2002, **11**:131–138.

159. Ickenroth MH, Grispen JE, Ronda G, Dinant G-J, de Vries NK, van der Weijden T: **Educating consumers in self-testing: The development of an online decision aid**. *Health Educ J* 2014, **74**:485–495.

160. Ronda G, Grispen JEJ, Ickenroth MHP, Dinant G-J, De Vries NK, Van der Weijden T: **The effects of a web-based decision aid on the intention to diagnostic self-testing for cholesterol and diabetes: a randomized controlled trial.** *BMC Public Health* 2014, **14**:921.

161. Irwin E, Arnold A, Whelan TJ, Reyno LM, Cranton P: **Offering a choice between two adjuvant chemotherapy regimens: A pilot study to develop a decision aid for women with breast cancer**. *Patient Educ Couns* 1999, **37**:283–291.

162. Jackson C, Cheater FM, Peacock R, Leask J, Trevena L: **Evaluating a web-based MMR decision aid to support informed decision-making by UK parents: A before-and-after feasibility study**. *Health Educ J* 2010, **69**:74–83.

163. Shourie S, Jackson C, Cheater FM, Bekker HL, Edlin R, Tubeuf S, Harrison W, McAleese E, Schweiger M, Bleasby B, Hammond L: **A cluster randomised controlled trial of a web based decision aid to support parents’ decisions about their child's Measles Mumps and Rubella (MMR) vaccination**. *Vaccine* 2013, **31**:6003–6010.

164. Tubeuf S, Edlin R, Shourie S, Cheater FM, Bekker H, Jackson C: **Cost effectiveness of a web-based decision aid for parents deciding about MMR vaccination: A three-arm cluster randomised controlled trial in primary care**. *Br J Gen Pract* 2014, **64**:e493–e499.

165. Jenkinson J, Wilson-Pauwels L, Jewett MA, Woolridge N: **Development of a hypermedia program designed to assist patients with localized prostate cancer in making treatment decisions**. *J Biocommun* 1998, **25**:2–11.

166. Jibaja-Weiss ML, Volk RJ, Friedman LC, Granchi TS, Neff NE, Spann SJ, Robinson EK, Aoki N, Robert Beck J: **Preliminary testing of a just-in-time, user-defined values clarification exercise to aid lower literate women in making informed breast cancer treatment decisions.** *Health Expect* 2006, **9**:218–231.

167. Jibaja-Weiss ML, Volk RJ, Granch TS, Nefe NE, Spann SJ, Aoki N, Robinson EK, Freidman LC, Beck JR: **Entertainment education for informed breast cancer treatment decisions in low-literate women: development and initial evaluation of a patient decision aid**. *J cancer Educ* 2006, **21**:133–139.

168. Jibaja-Weiss ML, Volk RJ, Granchi TS, Neff NE, Robinson EK, Spann SJ, Aoki N, Friedman LC, Beck JR: **Entertainment education for breast cancer surgery decisions: A randomized trial among patients with low health literacy**. *Patient Educ Couns* 2011, **84**:41–48.

169. Johnson BR, Schwartz A, Goldberg J, Koerber A: **A chairside aid for shared decision making in dentistry: a randomized controlled trial.** *J Dent Educ* 2006, **70**:133–41.

170. Johnson SL, Kim YM, Church K: **Towards client-centered counseling: Development and testing of the WHO Decision-Making Tool**. *Patient Educ Couns* 2010, **81**:355–361.

171. Langston AM, Rosario L, Westhoff CL: **Structured contraceptive counseling-A randomized controlled trial**. *Patient Educ Couns* 2010, **81**:362–367.

172. Juan AS, Wakefield C, Kasparian NA, Kirk J, Tyler J: **Development and pilot testing of a decision aid for men considering genetic testing for breast and/or ovarian cancer-related mutations (BRCA1/2)**. *Genetic Testing* 2008:523–532.

173. Juraskova I, Butow P, Lopez A, Seccombe M, Coates A, Boyle F, McCarthy N, Reaby L, Forbes JF: **Improving informed consent: Pilot of a decision aid for women invited to participate in a breast cancer prevention trial (IBIS-II DCIS)**. *Heal Expect* 2008, **11**:252–262.

174. Juraskova I, Butow P, Bonner C, Bell ML, Smith AB, Seccombe M, Boyle F, Reaby L, Cuzick J, Forbes JF: **Improving decision making about clinical trial participation - a randomised controlled trial of a decision aid for women considering participation in the IBIS-II breast cancer prevention trial.** *Br J Cancer* 2014, **111**:1–7.

175. Kellar I, Sutton S, Griffin S, Prevost AT, Kinmonth AL, Marteau TM: **Evaluation of an informed choice invitation for type 2 diabetes screening**. *Patient Educ Couns* 2008, **72**:232–238.

176. Mann E, Kellar I, Sutton S, Kinmonth AL, Hankins M, Griffin S, Marteau TM: **Impact of informed-choice invitations on diabetes screening knowledge, attitude and intentions: an analogue study**. *BMC Public Health* 2010, **10**:768.

177. Marteau TM, Mann E, Prevost a T, Vasconcelos JC, Kellar I, Sanderson S, Parker M, Griffin S, Sutton S, Kinmonth AL: **Impact of an informed choice invitation on uptake of screening for diabetes in primary care (DICISION): randomised trial**. *BMJ* 2010, **340**:c2138.

178. Kellar I, Mann E, Kinmonth AL, Prevost AT, Sutton S, Marteau TM: **Can informed choice invitations lead to inequities in intentions to make lifestyle changes among participants in a primary care diabetes screening programme? Evidence from a randomized trial**. *Public Health* 2011, **125**:645–652.

179. Kennedy ADM, Sculpher MJ, Coulter A, Rees M, Abrams KR, Horsley S, Cowley D, Kidson C, Kirwin C, Naish C: **Effects of decision aids for menorrhagia on treatment choices, health outcomes, and costs: a randomized controlled trial**. *JAMA* 2002, **288**:2701–2708.

180. Kennedy ADM, Sculpher MJ, Coulter A, Dwyer N, Rees M, Horsley S, Cowley D, Kidson C, Kirwin C, Naish C, Bidgood K, Cullimore J, Kerr-Wilson R, Abrams KR, Stirrat G: **A multicentre randomised controlled trial assessing the costs and benefits of using structured information and analysis of women’s preferences in the management of menorrhagia**. *Health Technology Assessment* 2003:1–76.

181. Kiatpongsan S, Carlson K, Feibelmann S, Sepucha K: **Decision aid reduces misperceptions about hormone therapy: a randomized controlled trial**. *Menopause* 2014, **21**:33–38.

182. Knapp P, Wanklyn P, Raynor DK, Waxman R: **Developing and testing a patient information booklet for thrombolysis used in acute stroke**. *Int J Pharm Pract* 2010, **18**:362–369.

183. Krist AH, Woolf SH, Johnson RE, Kerns JW: **Patient education on prostate cancer screening and involvement in decision making**. *Ann Fam Med* 2007, **5**:112–119.

184. Krones T, Keller H, Becker A, S??nnichsen A, Baum E, Donner-Banzhoff N: **The theory of planned behaviour in a randomized trial of a decision aid on cardiovascular risk prevention**. *Patient Educ Couns* 2010, **78**:169–176.

185. Kuppermann M, Pena S, Bishop JT, Nakagawa S, Gregorich SE, Sit A, Vargas J, Caughey AB, Sykes S, Pierce L, Norton ME: **Effect of Enhanced Information, Values Clarification, and Removal of Financial Barriers on Use of Prenatal Genetic Testing**. *JAMA* 2014, **312**:1210.

186. Norton M, Nakagawa S, Kuppermann M: **Women’s Attitudes Regarding Prenatal Testing for a Range of Congenital Disorders of Varying Severity**. *J Clin Med* 2014, **3**:144–152.

187. Labrecque M, Paunescu C, Plesu I, Stacey D, Legare F: **Evaluation of the effect of a patient decision aid about vasectomy on the decision-making process: a randomized trial**. *Contraception* 2010, **82**:556–562.

188. Lalonde L, O’Connor AM, Drake E, Duguay P, Lowensteyn I, Grover SA: **Development and preliminary testing of a patient decision aid to assist pharmaceutical care in the prevention of cardiovascular disease**. *Pharmacotherapy* 2004, **24**:909–922.

189. Lalonde L, O’Connor AM, Duguay P, Brassard J, Drake E, Grover SA: **Evaluation of a decision aid and a personal risk profile in community pharmacy for patients considering options to improve cardiovascular health: the OPTIONS pilot study**. *Int J Pharm Pract* 2006, **14**:51–62.

190. LaVista JM, Treise DM, Dunbar LN, Ritho J, Hartzema AG, Lottenberg R: **Development and evaluation of a patient empowerment video to promote hydroxyurea adoption in sickle cell disease.** *J Natl Med Assoc* 2009, **101**:251–7.

191. Lawrence VA, Streiner D, Hazuda HP, Naylor R, Levine M, Gafni A: **A cross-cultural consumer-based decision aid for screening mammography**. *Prev Med (Baltim)* 2000, **30**:200–208.

192. Legare F, Stacey D, Dodin S, O’Connor A, Richer M, Griffiths F, LeBlanc A, Rousseau JLC, Tapp S: **Women’s decision making about the use of natural health products at menopause: a needs assessment and patient decision aid**. *J Altern Complement Med* 2007, **13**:741–749.

193. Legare F, Dodin S, Stacey D, Leblanc A, Tapp S: **Patient decision aid on natural health products for menopausal symptoms: randomized controlled trial**. *Menopause Int* 2008, **14**:105–110.

194. Menard P, Stacey D, Legare F, Woodend K: **Evaluation of a natural health product decision aid: A tool for middle aged women considering menopausal symptom relief**. *Maturitas* 2010, **65**:366–371.

195. Legare F, Labrecque M, LeBlanc A, Njoya M, Laurier C, Côté L, Godin G, Thivierge RL, O’Connor A, St-Jacques S: **Training family physicians in shared decision making for the use of antibiotics for acute respiratory infections: a pilot clustered randomized controlled trial.** *Health Expect* 2011, **14**(Suppl 1):96–110.

196. Lerman C, Lustbader E, Rimer B, Daly M, Miller S, Sands C, Balshem A: **Effects of Individualized Breast Cancer Risk Counseling: a Randomized Trial**. *J Natl Cancer Inst* 1995, **87**:286 –292.

197. Lewis C, Pignone M, Schild LA, Scott T, Winquist A, Rimer BK, Glanz K: **Effectiveness of a Patient and Practice-Level Colorectal Cancer Screening Intervention in Health Plan Members: Design and Baseline Findings of the CHOICE Trial**. *Cancer* 2010, **116**:1664–1673.

198. Miller DP, Spangler JG, Case LD, Goff DC, Singh S, Pignone MP: **Effectiveness of a web-based colorectal cancer screening patient decision aid: A randomized controlled trial in a mixed-literacy population**. *Am J Prev Med* 2011, **40**:608–615.

199. Pignone M, Winquist A, Schild LA, Lewis C, Scott T, Hawley J, Rimer BK, Glanz K: **Effectiveness of a patient and practice-level colorectal cancer screening intervention in health plan members: The CHOICE trial**. *Cancer* 2011, **117**:3352–3362.

200. Li LC, Adam PM, Townsend AF, Lacaille D, Yousefi C, Stacey D, Gromala D, Shaw CD, Tugwell P, Backman CL: **Usability testing of ANSWER: a web-based methotrexate decision aid for patients with rheumatoid arthritis.** *BMC Med Inform Decis Mak* 2013, **13**:131.

201. Li LC, Adam PM, Backman CL, Lineker S, Jones CA, Lacaille D, Townsend AF, Yacyshyn E, Yousefi C, Tugwell P, Leese J, Stacey D: **A proof-of-concept study of ANSWER, a web-based methotrexate decision aid for patients with rheumatoid arthritis.** *Arthritis Care Res (Hoboken)* 2014, **66**:1472–1481.

202. Liao L, Jollis JG, DeLong ER, Peterson ED, Morris KG, Mark DB: **Impact of an interactive video on decision making of patients with ischemic heart disease.** *J Gen Intern Med* 1996, **11**:373–376.

203. Morgan MW, Deber RB, Llewellyn-Thomas HA, Gladstone P, Cusimano RJ, O’Rourke K, Tomlinson G, Detsky AS: **Randomized, controlled trial of an interactive videodisc decision aid for patients with ischemic heart disease**. *J Gen Intern Med* 2000, **15**:685–693.

204. Loh A, Simon D, Wills CE, Kriston L, Niebling W, Härter M: **The effects of a shared decision-making intervention in primary care of depression: A cluster-randomized controlled trial**. *Patient Educ Couns* 2007, **67**:324–332.

205. Lurie JD, Spratt KF, Blood EA, Tosteson TD, Tosteson ANA, Weinstein JN: **Effects of viewing an evidence-based video decision aid on patients’ treatment preferences for spine surgery**. *Spine (Phila Pa 1976)* 2011, **36**:1501–1504.

206. Mancini J, Nogu??s C, Adenis C, Berthet P, Bonadona V, Chompret A, Coupier I, Eisinger F, Fricker JP, Gauthier-Villars M, Lasset C, Lortholary A, N’Guyen TD, Vennin P, Sobol H, Stoppa-Lyonnet D, Julian-Reynier C: **Impact of an information booklet on satisfaction and decision-making about BRCA genetic testing**. *Eur J Cancer* 2006, **42**:871–881.

207. Man-Son-Hing M, Laupacis A, O’Connor AM, Biggs J, Drake E, Yetisir E, Hart RG: **A patient decision aid regarding antithrombotic therapy for stroke prevention in atrial fibrillation: a randomized controlled trial.** *JAMA* 1999, **282**:737–43.

208. Man-Son-Hing M, Laupacis A, O’Connor AM, Hart RG, Feldman G, Blackshear JL, Anderson DC: **Development of a decision aid for patients with atrial fibrillation who are considering antithrombotic therapy**. *J Gen Intern Med* 2000, **15**:723–730.

209. McAlister F a, Man-Son-Hing M, Straus SE, Ghali W a, Anderson D, Majumdar SR, Gibson P, Cox JL, Fradette M: **Impact of a patient decision aid on care among patients with nonvalvular atrial fibrillation: a cluster randomized trial.** *CMAJ* 2005, **173**:496–501.

210. Mathers N, Ng CJ, Campbell MJ, Colwell B, Brown I, Bradley A: **Clinical effectiveness of a patient decision aid to improve decision quality and glycaemic control in people with diabetes making treatment choices: a cluster randomised controlled trial (PANDAs) in general practice**. *BMJ Open* 2012, **2**:e001469.

211. Ng CJ, Mathers N, Bradley A, Colwell B: **A “combined framework” approach to developing a patient decision aid: the PANDAs model**. *BMC Health Serv Res* 2014, **14**:503.

212. Mathieu E, Barratt A, Davey HM, McGeechan K, Howard K, Houssami N: **Informed choice in mammography screening: a randomized trial of a decision aid for 70-year-old women.** *Arch Intern Med* 2007, **167**:2039–2046.

213. Mathieu E, Barratt AL, McGeechan K, Davey HM, Howard K, Houssami N: **Helping women make choices about mammography screening: An online randomized trial of a decision aid for 40-year-old women**. *Patient Educ Couns* 2010, **81**:63–72.

214. Matlock DD, Keech TAE, Mckenzie MB, Bronsert MR, Nowels CT, Kutner JS: **Feasibility and acceptability of a decision aid designed for people facing advanced or terminal illness: A pilot randomized trial**. *Heal Expect* 2014, **17**:49–59.

215. Mayer DK, Ratichek S, Berhe H, Stewart S, McTavish F, Gustafson D, Parsons SK: **Development of a health-related website for parents of children receiving hematopoietic stem cell transplant: HSCT-CHESS**. *J Cancer Surviv* 2010, **4**:67–73.

216. McCaffery KJ, Irwig L: **Australian women’s needs and preferences for information about human papillomavirus in cervical screening.** *J Med Screen* 2005, **12**:134–141.

217. McCaffery K, Waller J, Nazroo J, Wardle J: **Social and psychological impact of HPV testing in cervical screening: a qualitative study.** *Sex Transm Infect* 2006, **82**:169–74.

218. McCaffery KJ, Irwig L, Chan SF, Macaskill P, Barratt A, Lewicka M, Clarke J, Weisberg E: **HPV testing versus repeat Pap testing for the management of a minor abnormal Pap smear: Evaluation of a decision aid to support informed choice**. *Patient Educ Couns* 2008, **73**:473–481.

219. McCaffery KJ, Irwig L, Turner R, Chan SF, Macaskill P, Lewicka M, Clarke J, Weisberg E, Barratt A: **Psychosocial outcomes of three triage methods for the management of borderline abnormal cervical smears: an open randomised trial**. *BMJ* 2010, **340**:b4491.

220. McKay A, Martin W, Latosinsky S: **How should we inform women at higher risk of breast cancer about tamoxifen? An approach with a decision guide**. *Breast Cancer Res Treat* 2005, **94**:153–159.

221. Miller SM, Fleisher L, Roussi P, Buzaglo JS, Schnoll R, Slater E, Raysor S, Popa-Mabe M: **Facilitating informed decision making about breast cancer risk and genetic counseling among women calling the NCI’s Cancer Information Service**. *J Health Commun* 2005, **10**(Suppl 1):119–136.

222. Milne J, Gafni A, Lu D, Wood S, Sauve R, Ross S: **Developing and pre-testing a decision board to facilitate informed choice about delivery approach in uncomplicated pregnancy**. *BMC Pregnancy Childbirth* 2009, **9**:50.

223. Mitchell SL, Tetroe J, O’Connor AM: **A decision aid for long-term tube feeding in cognitively impaired older persons.** *J Am Geriatr Soc* 2001, **49**:313–6.

224. Hanson LC, Carey TS, Caprio AJ, Lee TJ, Ersek M, Garrett J, Jackman A, Gilliam R, Wessell K, Mitchell SL: **Improving decision-making for feeding options in advanced dementia: A randomized, controlled trial**. *J Am Geriatr Soc* 2011, **59**:2009–2016.

225. Snyder EA, Caprio AJ, Wessell K, Lin FC, Hanson LC: **Impact of a decision aid on surrogate decision-makers’ perceptions of feeding options for patients with dementia**. *J Am Med Dir Assoc* 2013, **14**:114–118.

226. Montgomery AA, Fahey T, Peters TJ: **A factorial randomised controlled trial of decision analysis and an information video plus leaflet for newly diagnosed hypertensive patients**. *Br J Gen Pract* 2003, **53**:446–453.

227. Emmett CL, Montgomery A a, Peters TJ, Fahey T: **Three-year follow-up of a factorial randomised controlled trial of two decision aids for newly diagnosed hypertensive patients.** *Br J Gen Pr* 2005, **55**:551–553.

228. Montori VM, Breslin M, Maleska M, Weymiller AJ: **Creating a conversation: Insights from the development of a decision aid**. *PLoS Medicine* 2007:1303–1307.

229. Weymiller AJ, Montori VM, Jones LA, Gafni A, Guyatt GH, Bryant SC, Christianson TJH, Mullan RJ, Smith SA: **Helping patients with type 2 diabetes mellitus make treatment decisions: statin choice randomized trial.** *Arch Intern Med* 2007, **167**:1076–82.

230. Jones LA, Weymiller AJ, Shah N, Bryant SC, Christianson TJH, Guyatt GH, Gafni A, Smith SA, Montori VM: **Should clinicians deliver decision aids? Further exploration of the statin choice randomized trial results.** *Med Decis Making* 2009, **29**:468–474.

231. Nannenga MR, Montori VM, Weymiller AJ, Smith SA, Christianson TJH, Bryant SC, Gafni A, Charles C, Mullan RJ, Jones LA, Bolona ER, Guyatt GH: **A treatment decision aid may increase patient trust in the diabetes specialist. the Statin Choice randomized trial**. *Heal Expect* 2009, **12**:38–44.

232. Mann DM, Ponieman D, Montori VM, Arciniega J, McGinn T: **The Statin Choice decision aid in primary care: A randomized trial**. *Patient Educ Couns* 2010, **80**:138–140.

233. Murray E, Davis H, Tai SS, Coulter A, Gray A, Haines A: **Randomised controlled trial of an interactive multimedia decision aid on benign prostatic hypertrophy in primary care.** *BMJ* 2001, **323**:493–6.

234. Murray E, Davis H, Tai SS, Coulter A, Gray A, Haines A: **Randomised controlled trial of an interactive multimedia decision aid on hormone replacement therapy in primary care.** *BMJ* 2001, **323**:490–3.

235. Nassar N, Roberts CL, Raynes-Greenow CH, Barratt A: **Development and pilot-testing of a decision aid for women with a breech-presenting baby**. *Midwifery* 2007, **23**:38–47.

236. Nassar N, Roberts CL, Raynes-Greenow CH, Barratt A, Peat B: **Evaluation of a decision aid for women with breech presentation at term: A randomised controlled trial [ISRCTN14570598]**. *BJOG* 2007, **114**:325–333.

237. Nozaki K, Okubo C, Yokoyama Y, Morita A, Akamatsu R, Nakayama T, Fukuhara S, Hashimoto N: **Examination of the effectiveness of DVD decision support tools for patients with unruptured cerebral aneurysms.** *Neurol Med Chir (Tokyo)* 2007, **47**:531–6.

238. O’Connor AM, Tugwell P, Wells GA, Elmslie T, Jolly E, Hollingworth G, Mcpherson R, Drake E, Hopman W, Mackenzie T: **Randomized trial of a portable, self-administered decision aid for postmenopausal women considering long-term preventive hormone therapy**. *Med Decis Mak* 1998, **18**:295–303.

239. O’Connor AM, Tugwell P, Wells GA, Elmslie T, Jolly E, Hollingworth G, McPherson R, Bunn H, Graham I, Drake E: **A decision aid for women considering hormone therapy after menopause: Decision support framework and evaluation**. *Patient Educ Couns* 1998, **33**:267–279.

240. Rostom A, O’Connor A, Tugwell P, Wells G: **A randomized trial of a computerized versus an audio-booklet decision aid for women considering post-menopausal hormone replacement therapy**. *Patient Educ Couns* 2002, **46**:67–74.

241. Onel E, Hamond C, Wasson JH, Berlin BB, Ely MG, Laudone VP, Tarantino AE, Albertsen PC: **Assessment of the feasibility and impact of shared decision making in prostate cancer**. *Urology* 1998, **51**:63–66.

242. Ozanne EM, Annis C, Adduci K, Showstack J, Esserman L: **Pilot Trial of a Computerized Decision Aid for Breast Cancer Prevention**. *Breast J* 2007, **13**:147–154.

243. Peate M, Meiser B, Friedlander M, Saunders C, Martinello R, Wakefield CE, Hickey M: **Development and Pilot Testing of a Fertility Decision Aid for Young Women Diagnosed with Early Breast Cancer**. *Breast J* 2011, **17**:112–114.

244. Peate M, Meiser B, Friedlander M, Zorbas H, Rovelli S, Sansom-Daly U, Sangster J, Hadzi-Pavlovic D, Hickey M: **It’s now or never: Fertility-related knowledge, decision-making preferences, and treatment intentions in young women with breast cancer - An Australian fertility decision aid collaborative group study**. *J Clin Oncol* 2011, **29**:1670–1677.

245. Peate M, Meiser B, Cheah BC, Saunders C, Butow P, Thewes B, Hart R, Phillips K, Hickey M, Friedlander M: **Making hard choices easier: a prospective, multicentre study to assess the efficacy of a fertility-related decision aid in young women with early-stage breast cancer.** *Br J Cancer* 2012, **106**:1053–61.

246. Pencille LJ, Campbell ME, Van Houten HK, Shah ND, Mullan RJ, Swiglo BA, Breslin M, Kesman RL, Tulledge-Scheitel SM, Jaeger TM, Johnson RE, Bartel GA, Wermers RA, Melton LJ, Montori VM: **Protocol for the Osteoporosis Choice trial. A pilot randomized trial of a decision aid in primary care practice**. *Trials* 2009, **10**:113.

247. Montori VM, Shah ND, Pencille LJ, Branda ME, Van Houten HK, Swiglo BA, Kesman RL, Tulledge-Scheitel SM, Jaeger TM, Johnson RE, Bartel GA, Melton LJ, Wermers RA: **Use of a decision aid to improve treatment decisions in osteoporosis: The osteoporosis choice randomized trial**. *Am J Med* 2011, **124**:549–556.

248. Perestelo-Perez L, Perez-Ramos J, Gonzalez-Lorenzo M, Rivero-Santana A, Serrano-Aguilar P: **Decision aids for patients facing health treatment decisions in Spain: preliminary results.** *Patient Educ Couns* 2010, **80**:364–372.

249. Permuth-Wey J, Vadaparampil S, Rumphs A, Kinney AY, Pal T: **Development of a culturally tailored genetic counseling booklet about hereditary breast and ovarian cancer for black women**. *Am J Med Genet Part A* 2010, **152**:836–845.

250. Pierce MA, Hess EP, Kline JA, Shah ND, Breslin M, Branda ME, Pencille LJ, Asplin BR, Nestler DM, Sadosty AT, Stiell IG, Ting HH, Montori VM: **The Chest Pain Choice trial: a pilot randomized trial of a decision aid for patients with chest pain in the emergency department**. *Trials* 2010, **11**:57.

251. Hess EP, Knoedler MA, Shah ND, Kline JA, Breslin M, Branda ME, Pencille LJ, Asplin BR, Nestler DM, Sadosty AT, Stiell IG, Ting HH, Montori VM: **The chest pain choice decision aid: A randomized trial**. *Circ Cardiovasc Qual Outcomes* 2012, **5**:251–259.

252. Pignone M, Harris R, Kinsinger L: **Videotape-based decision aid for colon cancer screening. A randomized, controlled trial**. *Ann Intern Med* 2000, **133**:761–769.

253. Kim J, Whitney A, Hayter S, Lewis C, Campbell M, Sutherland L, Fowler B, Googe S, McCoy R, Pignone M: **Development and initial testing of a computer-based patient decision aid to promote colorectal cancer screening for primary care practice**. *BMC Med Inform Decis Mak* 2005, **5**:36.

254. Griffith JM, Fichter M, Fowler FJ, Lewis C, Pignone MP: **Should a colon cancer screening decision aid include the option of no testing? A comparative trial of two decision aids**. *BMC Med Inform Decis Mak* 2008, **8**:10.

255. Griffith JM, Lewis CL, Brenner ART, Pignone MP: **The effect of offering different numbers of colorectal cancer screening test options in a decision aid: a pilot randomized trial**. *BMC Med Inform Decis Mak* 2008, **8**:4.

256. Pignone M, Sheridan SL, Lee YZ, Kuo J, Phillips C, Mulrow C, Zeiger R: **Heart to Heart: a computerized decision aid for assessment of coronary heart disease risk and the impact of risk-reduction interventions for primary prevention.** *Prev Cardiol* 2004, **7**:26–33.

257. Sheridan SL, Shadle J, Simpson RJ, Pignone MP: **The impact of a decision aid about heart disease prevention on patients’ discussions with their doctor and their plans for prevention: a pilot randomized trial**. *BMC Health Serv Res* 2006, **6**:121.

258. Sheridan SL, Turner A, Pignone MP, Fowler B, Kibe J, Carr C, Behrend L, Keyserling T, Simpson RJJ, Rimer B: **The Development of a Comprehensive Intervention to Improve Decision-making and Adherence to Coronary Heart Disease Risk Reducing Strategies: Heart to Heart 2**. *Patient Educ Couns* 2009, **in press**.

259. Sheridan SL, Griffith JM, Behrend L, Gizlice Z, Jianwen Cai, Pignone MP: **Effect of adding a values clarification exercise to a decision aid on heart disease prevention: a randomized trial**. *Med Decis Mak* 2010, **30**:E28–39.

260. Sheridan SL, Draeger LB, Pignone MP, Keyserling TC, Simpson Jr. RJ, Rimer B, Bangdiwala SI, Cai J, Gizlice Z: **A randomized trial of an intervention to improve use and adherence to effective coronary heart disease prevention strategies**. *BMC Health Serv Res* 2011, **11**:1–10.

261. Raats CJI, van Veenendaal H, Versluijs MM, Burgers JS: **A generic tool for development of decision aids based on clinical practice guidelines.** *Patient Educ Couns* 2008, **73**:413–7.

262. Raynes-Greenow CH, Roberts CL, Nassar N, Trevena L: **Do audio-guided decision aids improve outcomes? A randomized controlled trial of an audio-guided decision aid compared with a booklet decision aid for Australian women considering labour analgesia**. *Heal Expect* 2009, **12**:407–416.

263. Raynes-Greenow CH, Nassar N, Torvaldsen S, Trevena L, Roberts CL: **Assisting informed decision making for labour analgesia: a randomised controlled trial of a decision aid for labour analgesia versus a pamphlet**. *BMC Pregnancy Childbirth* 2010, **10**:15.

264. Rothert ML, Holmes-Rovner M, Rovner D, Kroll J, Breer L, Talarczyk G, Schmitt N, Padonu G, Wills C: **An educational intervention as decision support for menopausal women.** *Res Nurs Health* 1997, **20**:377–87.

265. Holmes-Rovner M, Kroll J, Rovner DR, Schmitt N, Rothert M, Padonu G, Talarczyk G: **Patient decision support intervention: increased consistency with decision analytic models.** *Med Care* 1999, **37**:270–284.

266. Ruthman JL, Ferrans CE: **Efficacy of a video for teaching patients about prostate cancer screening and treatment**. *Am J Heal Promot* 2004, **18**:292–295.

267. Saver BG, Gustafson D, Taylor TR, Hawkins RP, Woods NF, Dinauer S, Casey S, MacLaren-Loranger A: **A tale of two studies: The importance of setting, subjects and context in two randomized, controlled trials of a web-based decision support for perimenopausal and postmenopausal health decisions**. *Patient Educ Couns* 2007, **66**:211–222.

268. Sawka C a, Goel V, Mahut C a, Taylor G a, Thiel EC, O’Connor a M, Ackerman I, Burt JH, Gort EH: **Development of a patient decision aid for choice of surgical treatment for breast cancer**. *Heal Expect* 1998, **1**:23–36.

269. Goel V, Sawka C a, Thiel EC, Gort EH, O’Connor a M: **Randomized trial of a patient decision aid for choice of surgical treatment for breast cancer**. *Med Decis Mak* 2001, **21**:1–6.

270. Schackmann EA, Munoz DF, Mills MA, Plevritis SK, Kurian AW: **Feasibility evaluation of an online tool to guide decisions for BRCA1/2 mutation carriers**. *Fam Cancer* 2013, **12**:65–73.

271. Schapira MM, VanRuiswyk J: **The effect of an illustrated pamphlet decision-aid on the use of prostate cancer screening tests.** *J Fam Pract* 2000, **49**:418–24.

272. Schapira MM, Meade C, Nattinger AB: **Enhanced decision-making: The use of a videotape decision-aid for patients with prostate cancer**. *Patient Educ Couns* 1997, **30**:119–127.

273. Schapira MM, Gilligan MA, McAuliffe T, Garmon G, Carnes M, Nattinger AB: **Decision-making at menopause: A randomized controlled trial of a computer-based hormone therapy decision-aid**. *Patient Educ Couns* 2007, **67**:100–107.

274. Schonberg MA, Hamel MB, Davis RB, Griggs MC, Wee CC, Fagerlin A, Marcantonio ER: **Development and evaluation of a decision aid on mammography screening for women 75 years and older.** *JAMA Intern Med* 2014, **174**:417–24.

275. Schroy PC, Emmons K, Peters E, Glick JT, Robinson PA, Lydotes MA, Mylvanaman S, Evans S, Chaisson C, Pignone M, Prout M, Davidson P, Heeren TC: **The impact of a novel computer-based decision aid on shared decision making for colorectal cancer screening: a randomized trial.** *Med Decis Making* 2011, **31**:93–107.

276. Schroy PC, Emmons KM, Peters E, Glick JT, Robinson PA, Lydotes MA, Mylvaganam SR, Coe AM, Chen CA, Chaisson CE, Pignone MP, Prout MN, Davidson PK, Heeren TC: **Aid-assisted decision making and colorectal cancer screening: A randomized controlled trial**. *Am J Prev Med* 2012, **43**:573–583.

277. Schroy PC, Mylvaganam S, Davidson P: **Provider perspectives on the utility of a colorectal cancer screening decision aid for facilitating shared decision making**. *Heal Expect* 2014, **17**:27–35.

278. Schwalm JD, Stacey D, Pericak D, Natarajan MK: **Radial artery versus femoral artery access options in coronary angiogram procedures: Randomized controlled trial of a patient-decision aid**. *Circ Cardiovasc Qual Outcomes* 2012, **5**:260–266.

279. Schwartz LM, Woloshin S, Welch HG: **Using a drug facts box to communicate drug benefits and harms: Two randomized trials**. *Ann Intern Med* 2009, **150**:516–527.

280. Schwartz MD, Valdimarsdottir HB, DeMarco TA, Peshkin BN, Lawrence W, Rispoli J, Brown K, Isaacs C, O’Neill S, Shelby R, Grumet SC, McGovern MM, Garnett S, Bremer H, Leaman S, O’Mara K, Kelleher S, Komaridis K: **Randomized trial of a decision aid for BRCA1/BRCA2 mutation carriers: impact on measures of decision making and satisfaction.** *Health Psychol* 2009, **28**:11–9.

281. Sepucha KR, Ozanne EM, Partridge AH, Moy B: **Is there a role for decision aids in advanced breast cancer?** *Med Decis Making* 2009, **29**:475–82.

282. Shaffer VA, Owens J, Zikmund-Fisher BJ: **The Effect of patient narratives on information search in a web-based breast cancer decision aid: An eye-tracking study**. *J Med Internet Res* 2013, **15**:e273.

283. Sheppard VB, Williams KP, Harrison TM, Jennings Y, Lucas W, Stephen J, Robinson D, Mandelblatt JS, Taylor KL: **Development of decision-support intervention for Black women with breast cancer**. *Psychooncology* 2010, **19**:62–70.

284. Sheridan SL, Felix K, Pignone MP, Lewis CL: **Information needs of men regarding prostate cancer screening and the effect of a brief decision aid**. *Patient Educ Couns* 2004, **54**:345–351.

285. Sherman KA, Harcourt DM, Lam TC, Shaw LK, Boyages J: **BRECONDA: Development and acceptability of an interactive decisional support tool for women considering breast reconstruction**. *Psychooncology* 2014, **23**:835–838.

286. Shorten A, Chamberlain M, Shorten B, Kariminia A: **Making choices for childbirth: Development and testing of a decision-aid for women who have experienced previous caesarean**. *Patient Educ Couns* 2004, **52**:307–313.

287. Shorten A, Shorten B, Keogh J, West S, Morris J: **Making Choices for Childbirth: A Randomized Controlled Trial of a Decision‐aid for Informed Birth after Cesarean**. *Birth* 2005, **32**:252–261.

288. Simon D, Kriston L, Von Wolff A, Buchholz A, Vietor C, Hecke T, Loh A, Zenker M, Weiss M, Härter M: **Effectiveness of a web-based, individually tailored decision aid for depression or acute low back pain: A randomized controlled trial**. *Patient Educ Couns* 2012, **87**:360–368.

289. Smith SK, Trevena L, Nutbeam D, Barratt A, McCaffery KJ: **Information needs and preferences of low and high literacy consumers for decisions about colorectal cancer screening: Utilizing a linguistic model**. *Heal Expect* 2008, **11**:123–136.

290. Trevena LJ, Irwig L, Barratt A: **Randomized trial of a self-administered decision aid for colorectal cancer screening.** *J Med Screen* 2008, **15**:76–82.

291. Smith SK, Trevena L, Barratt A, Dixon A, Nutbeam D, Simpson JM, McCaffery KJ: **Development and preliminary evaluation of a bowel cancer screening decision aid for adults with lower literacy**. *Patient Educ Couns* 2009, **75**:358–367.

292. Smith SK, Trevena L, Simpson JM, Barratt A, Nutbeam D, McCaffery KJ: **A decision aid to support informed choices about bowel cancer screening among adults with low education: randomised controlled trial**. *BMJ* 2010, **341**:c5370.

293. Solberg LI, Asche SE, Sepucha K, Thygeson NM, Madden JE, Morrissey L, Kraemer KK, Anderson LH: **Informed choice assistance for women making uterine fibroid treatment decisions: a practical clinical trial.** *Med Decis Making* 2010, **30**:444–52.

294. Spunt BS, Deyo RA, Taylor VM, Leek KM, Goldberg HI, Mulley AG: **An interactive videodisc program for low back pain patients**. *Health Educ Res* 1996, **11**:535–541.

295. Deyo R a, Cherkin DC, Weinstein J, Howe J, Ciol M, Mulley a G: **Involving patients in clinical decisions: impact of an interactive video program on use of back surgery.** *Med Care* 2000, **38**:959–969.

296. Phelan EA, Deyo RA, Cherkin DC, Weinstein JN, Ciol MA, Kreuter W, Howe JF: **Helping patients decide about back surgery: a randomized trial of an interactive video program**. *Spine (Phila Pa 1976)* 2001, **26**:206–212.

297. Sridhar A, Chen A, Forbes ER, Glik D: **Mobile application for information on reversible contraception: a randomized controlled trial**. *Am J Obstet Gynecol* 2015, **212**:774–e1.

298. Stacey D, O’Connor AM, DeGrasse C, Verma S: **Development and evaluation of a breast cancer prevention decision aid for higher-risk women.** *Health Expect* 2003, **6**:3–18.

299. Stacey D, Hawker G, Dervin G, Tugwell P, Boland L, Pomey M-P, O’Connor AM, Taljaard M: **Decision aid for patients considering total knee arthroplasty with preference report for surgeons: a pilot randomized controlled trial**. *BMC Musculoskelet Disord* 2014, **15**:54.

300. Stalmeier PFM, Roosmalen MS: **Concise evaluation of decision aids.** *Patient Educ Couns* 2009, **74**:104–109.

301. Steckelberg A, Kasper J, Redegeld M, Mühlhauser I: **Risk information--barrier to informed choice? A focus group study.** *Soz Praventivmed* 2004, **49**:375–80.

302. Steckelberg A, Hülfenhaus C, Haastert B, Mühlhauser I: **Effect of evidence based risk information on “informed choice” in colorectal cancer screening: randomised controlled trial**. *BMJ* 2011, **342**:d3193.

303. Stein RA, Sharpe L, Bell ML, Boyle FM, Dunn SM, Clarke SJ: **Randomized controlled trial of a structured intervention to facilitate end-of-life decision making in patients with advanced cancer.** *J Clin Oncol* 2013, **31**:3403–3410.

304. Stiggelbout AM, Molewijk AC, Otten W, Van Bockel JH, Bruijninckx CM a, Van der Salm I, Kievit J: **The impact of individualized evidence-based decision support on aneurysm patients’ decision making, ideals of autonomy, and quality of life.** *Med Decis Making* 2008, **28**:751–62.

305. Stirling C, Leggett S, Lloyd B, Scott J, Blizzard L, Quinn S, Robinson A: **Decision aids for respite service choices by carers of people with dementia: development and pilot RCT.** *BMC Med Inform Decis Mak* 2012, **12**:21.

306. Sudore RL, Knight SJ, McMahan RD, Feuz M, Farrell D, Miao Y, Barnes DE: **A novel website to prepare diverse older adults for decision making and advance care planning: A pilot study**. *J Pain Symptom Manage* 2014, **47**:674–686.

307. Tan J, Wolfe B: **A patient decision aid for psoriasis based on current clinical practice guidelines.** *Arch Dermatol* 2012, **148**:718–23.

308. Tan J, Wolfe B: **Improved decisional conflict and preparedness for decision making using a patient decision aid for treatment selection in psoriasis: a pilot study.** *J Cutan Med Surg* 2014, **18**:114.

309. Dorfman CS, Williams RM, Kassan EC, Red SN, Dawson DL, Tuong W, Parker ER, Ohene-Frempong J, Davis KM, Krist AH, Woolf SH, Schwartz MD, Fishman MB, Cole C, Taylor KL: **The development of a web- and a print-based decision aid for prostate cancer screening**. *BMC Med Inform Decis Mak* 2010, **10**:12.

310. Kassan EC, Williams RM, Kelly SP, Barry SA, Penek S, Fishman MB, Cole CA, Miller EM, Taylor KL: **Men’s Use of an Internet-Based Decision Aid for Prostate Cancer Screening**. *J Health Commun* 2012, **17**:677–697.

311. Taylor KL, Williams RM, Davis K, Luta G, Penek S, Barry S, Kelly S, Tomko C, Schwartz M, Krist AH, Woolf SH, Fishman MB, Cole C, Miller E: **Decision Making in Prostate Cancer Screening Using Decision Aids vs Usual Care: A Randomized Clinical Trial**. *JAMA Intern Med* 2013, **173**:1704–1712.

312. Williams RM, Davis KM, Luta G, Edmond SN, Dorfman CS, Schwartz MD, Lynch J, Ahaghotu C, Taylor KL: **Fostering informed decisions: A randomized controlled trial assessing the impact of a decision aid among men registered to undergo mass screening for prostate cancer**. *Patient Educ Couns* 2013, **91**:329–336.

313. Thomson R, Robinson A, Greenaway J, Lowe P: **Development and description of a decision analysis based decision support tool for stroke prevention in atrial fibrillation**. *Qual Saf Health Care* 2002, **11**:25–31.

314. Kaner E, Heaven B, Rapley T, Murtagh M, Graham R, Thomson R, May C: **Medical communication and technology: a video-based process study of the use of decision aids in primary care consultations**. *BMC Med Inform Decis Mak* 2007, **7**:2.

315. Thomson RG, Eccles MP, Steen IN, Greenaway J, Stobbart L, Murtagh MJ, May CR: **A patient decision aid to support shared decision-making on anti-thrombotic treatment of patients with atrial fibrillation: randomised controlled trial.** *Qual Saf Health Care* 2007, **16**:216–223.

316. Tiller K, Meiser B, Reeson E, Tucker M, Andrews L, Gaff C, Kirk J, Phillips KA, Friedlander M: **A decision aid for women at increased risk for ovarian cancer**. *Int J Gynecol CANCER* 2003, **13**:15–22.

317. Tiller K, Meiser B, Gaff C, Kirk J, Dudding T, Phillips K, Friedlander M, Tucker K: **A randomized controlled trial of a decision aid for women at increased risk of ovarian cancer.** *Med Decis Making* 2006, **26**:360–72.

318. van der Krieke L, Emerencia AC, Aiello M, Sytema S: **Usability evaluation of a web-based support system for people with a schizophrenia diagnosis**. *J Med Internet Res* 2012, **14**:e24.

319. van Peperstraten AM, Hermens RPMG, Nelen WLDM, Stalmeier PFM, Wetzels AMM, Maas PHM, Kremer JAM, Grol RPTM: **Deciding how many embryos to transfer after in vitro fertilisation: Development and pilot test of a decision aid**. *Patient Educ Couns* 2010, **78**:124–129.

320. van Peperstraten A, Nelen W, Grol R, Zielhuis G, Adang E, Stalmeier P, Hermens R, Kremer J: **The effect of a multifaceted empowerment strategy on decision making about the number of embryos transferred in in vitro fertilisation: randomised controlled trial**. *BMJ* 2010, **341**:c2501.

321. van Tol-Geerdink JJ, Stalmeier PFM, van Lin ENJT, Schimmel EC, Huizenga H, van Daal WAJ, Leer JW: **Do prostate cancer patients want to choose their own radiation treatment?** *Int J Radiat Oncol Biol Phys* 2006, **66**:1105–1111.

322. van Tol-Geerdink JJ, Leer JW, van Lin ENJT, Schimmel EC, Huizenga H, van Daal WAJ, Stalmeier PFM: **Offering a Treatment Choice in the Irradiation of Prostate Cancer Leads to Better Informed and More Active Patients, Without Harm to Well-Being**. *Int J Radiat Oncol Biol Phys* 2008, **70**:442–448.

323. Van Tol-Geerdink JJ, Willem Leer J, Weijerman PC, Van Oort IM, Vergunst H, Van Lin EN, Alfred Witjes J, Stalmeier PF: **Choice between prostatectomy and radiotherapy when men are eligible for both: A randomized controlled trial of usual care vs decision aid**. *BJU Int* 2013, **111**:564–573.

324. Vandemheen KL, O’Connor A, Bell SC, Freitag A, Bye P, Jeanneret A, Berthiaume Y, Brown N, Wilcox P, Ryan G, Brager N, Rabin H, Morrison N, Gibson P, Jackson M, Paterson N, Middleton P, Aaron SD: **Randomized trial of a decision aid for patients with cystic fibrosis considering lung transplantation**. *Am J Respir Crit Care Med* 2009, **180**:761–768.

325. Vandemheen KL, Aaron SD, Poirier C, Tullis E, O’Connor A: **Development of a decision aid for adult cystic fibrosis patients considering referral for lung transplantation.** *Prog Transplant* 2010, **20**:81–87.

326. Volandes AE, Paasche-Orlow MK, Barry MJ, Gillick MR, Minaker KL, Chang Y, Cook EF, Abbo ED, El-Jawahri A, Mitchell SL: **Video decision support tool for advance care planning in dementia: randomised controlled trial**. *BMJ* 2009, **338**:b2159.

327. Volandes AE, Mitchell SL, Gillick MR, Chang Y, Paasche-Orlow MK: **Using Video Images to Improve the Accuracy of Surrogate Decision-Making: A Randomized Controlled Trial**. *J Am Med Dir Assoc* 2009, **10**:575–580.

328. Volandes AE, Barry MJ, Chang Y, Paasche-Orlow MK: **Improving decision making at the end of life with video images.** *Med Decis Making* 2010, **30**:29–34.

329. Volk RJ, Cass a R, Spann SJ: **A randomized controlled trial of shared decision making for prostate cancer screening.** *Arch Fam Med* 1999, **8**:333–40.

330. Volk RJ, Spann SJ, Cass AR, Hawley ST: **Patient education for informed decision making about prostate cancer screening: a randomized controlled trial with 1-year follow-up.** *Ann Fam Med* 2003, **1**:22–28.

331. Volk RJ, Jibaja-Weiss ML, Hawley ST, Kneuper S, Spann SJ, Miles BJ, Hyman DJ: **Entertainment education for prostate cancer screening: A randomized trial among primary care patients with low health literacy**. *Patient Educ Couns* 2008, **73**:482–489.

332. Volk RJ, Linder SK, Leal VB, Rabius V, Cinciripini PM, Kamath GR, Munden RF, Bevers TB: **Feasibility of a patient decision aid about lung cancer screening with low-dose computed tomography**. *Prev Med (Baltim)* 2014, **62**:60–63.

333. Wakefield CE, Meiser B, Homewood J, Peate M, Taylor A, Lobb E, Kirk J, Young MA, Williams R, Dudding T, Tucker K, Barlow-Stewart K, Fenton G, Goodwin A, Zodgekar P, Andrews L, Koeler J, Overkov A, Tyler J, Warner B, Gleeson M, Groombridge C, O’Donnell S, Spigelman A, McMahon C, Hossack L, Kentwell M, Aragona C, D’Souza R, Gaff C, et al.: **A randomized controlled trial of a decision aid for women considering genetic testing for breast and ovarian cancer risk**. *Breast Cancer Res Treat* 2008, **107**:289–301.

334. Wakefield CE, Watts KJ, Meiser B, Sansom-Daly U, Barratt A, Mann GJ, Lobb EA, Gaff CL, Howard K, Patel MI: **Development and pilot testing of an online screening decision aid for men with a family history of prostate cancer**. *Patient Educ Couns* 2011, **83**:64–72.

335. Watts KJ, Meiser B, Wakefield CE, Barratt AL, Howard K, Cheah BC, Mann GJ, Lobb EA, Gaff CL, Patel MI: **Online Prostate Cancer Screening Decision Aid for At-Risk Men: A Randomized Trial**. *Heal Psychol* 2014, **33**:986–997.

336. Weng HH, Kaplan RM, Boscardin WJ, Maclean CH, Lee IY, Chen W, Fitzgerald JD: **Development of a decision aid to address racial disparities in utilization of knee replacement surgery.** *Arthritis Rheum* 2007, **57**:568–575.

337. Willemsen MC, Wiebing M, Van Emst A, Zeeman G: **Helping smokers to decide on the use of efficacious smoking cessation methods: A randomized controlled trial of a decision aid**. *Addiction* 2006, **101**:441–449.

338. Wilson ME, Krupa A, Hinds RF, Litell JM, Swetz KM, Akhoundi A, Kashyap R, Gajic O, Kashani K: **A Video to Improve Patient and Surrogate Understanding of Cardiopulmonary Resuscitation Choices in the ICU: A Randomized Controlled Trial**. *Crit Care Med* 2015, **43**:621–9.

339. Wong SSM, Thornton JG, Gbolade B, Bekker HL: **A randomised controlled trial of a decision-aid leaflet to facilitate women’s choice between pregnancy termination methods**. *BJOG* 2006, **113**:688–694.

340. Wong J, D’Alimonte L, Angus J, Paszat L, Metcalfe K, Whelan T, Llewellyn-Thomas H, Warner E, Franssen E, Szumacher E: **Development of patients’ decision aid for older women with stage i breast cancer considering radiotherapy after lumpectomy**. *Int J Radiat Oncol Biol Phys* 2012, **84**:30–38.

341. Wright P, John C, Belt S: **Designing an interactive decision explorer**. *Information Design Journal* 2002:252–260.

342. Wright P, Belt S, John C: **Helping people assess the health risks from lifestyle choices: comparing a computer decision aid with customized printed alternative.** *Commun Med* 2004, **1**:183–192.

343. Zapka JG, Lemon SC, Puleo E, Estabrook B, Luckmann R, Erban S: **Patient education for colon cancer screening: A randomized trial of a video mailed before a physical examination**. *Ann Intern Med* 2004, **141**:683–692.

**Appendix 3. Variables Considered**

In this table, we use the term users as shorthand for patients, caregivers, family members, or surrogate decision makers, whichever was appropriate for the decision aid under consideration. Clinician users refers to clinicians who were not on the research team and who might potentially use the decision aid with their patients.

| **Variable** | **Projects that specifically involved members of vulnerable populations (n=30)** | **Projects that did not specifically involve members of vulnerable populations (n=157)** |
| --- | --- | --- |
| Decision aid is intended to be used at home or in a private setting | 13 (43%) | 85 (54%) |
| Decision aid is intended to be used in a health care setting but not during a clinical encounter | 8 (27%) | 47 (30%) |
| Decision aid is intended to be used during a clinical encounter | 9 (30%) | 61 (39%) |
| Decision aid users were involved in a formal needs assessment | 5 (17%) | 24 (15%) |
| **Decision aid users were involved in an informal needs assessment | 22 (73%) | 63 (40%) |
| *Decision aid users were involved in a content review prior to prototype development | 9 (30%) | 25 (16%) |
| Decision aid users were involved in prototype development | 7 (23%) | 38 (24%) |
| Decision aid users were involved in a content format review after prototype development | 11 (37%) | 57 (36%) |
| *Decision aid users were involved in a pilot test | 26 (87%) | 111 (71%) |
| Decision aid users were involved in a second pilot test | 3 (10%) | 17 (11%) |
| Decision aid development was iterative | 24 (80%) | 134 (85%) |
| Number of iterative cycles: Median (Interquartile range, full range) | 2.5 (0-3, 0-20) | 2 (0-3, 0-50) |
| Authors described what changes were made between versions/prototypes | 8 (27%) | 39 (25%) |
| *Developers asked users their thoughts & opinions of the tool | 27 (90%) | 117 (75%) |
| Developers asked clinician users their thoughts & opinions of the tool | 12 (40%) | 71 (45%) |
| Developers asked non-users (e.g., context experts, steering committee) their thoughts & opinions of the tool | 9 (30%) | 33 (21%) |
| Decision aid users were observed using the tool (not just asked their opinion) | 10 (33%) | 71 (45%) |
| *Developers assessed the impact of the decision aid on users | 25 (83%) | 146 (93%) |
| Users were clearly involved directly on the research/development team | 5 (17%) | 16 (10%) |
| *An advisory panel of users was involved in the project | 8 (27%) | 16 (10%) |
| Decision aid users were recruited as a convenience sample | 23 (77%) | 129 (82%) |
| **Decision aid users were recruited through community-based organizations | 12 (40%) | 17 (11%) |
| Decision aid users were recruited using flyers and posters | 4 (13%) | 19 (12%) |
| Decision aid users were recruited using registries | 5 (17%) | 24 (15%) |
| *Decision aid users were compensated/incentivized in some way | 16 (53%) | 54 (34%) |
| Clinician users were involved in the development process | 25 (83%) | 121 (77%) |
| Clinician users were consulted before an initial prototype was developed | 15 (50%) | 87 (57%) |
| Clinician users were consulted between the initial and final prototypes | 5 (17%) | 18 (11%) |
| Clinician users were consulted after the final prototype was developed | 19 (63%) | 88 (56%) |
| *An expert panel of academics, clinicians, etc. was involved in the project | 22 (73%) | 90 (57%) |
| *The project team had formal links with a specific patient or consumer organization | 7 (23%) | 21 (13%) |

*Variable entered into logistic regression.

**Variable significantly associated with dependent variable in logistic regression.
